# The importance of the traditional *milpa* in food security and nutritional self-sufficiency in the highlands of Oaxaca, Mexico

**DOI:** 10.1371/journal.pone.0246281

**Published:** 2021-02-19

**Authors:** Ivan P. Novotny, Pablo Tittonell, Mariela H. Fuentes-Ponce, Santiago López-Ridaura, Walter A. H. Rossing

**Affiliations:** 1 Doctorado en Ciencias Biológicas y de la Salud, Universidad Autónoma Metropolitana, Mexico, Mexico; 2 Farming Systems Ecology, Plant Sciences Group, Wageningen University and Research, Wageningen, Netherlands; 3 Agroecology, Environment and Systems Group, Instituto de Investigaciones Forestales y Agropecuarias de Bariloche (IFAB), INTA-CONICET, Bariloche, Argentina; 4 Agroécologie et Intensification Durable (AïDA), Centre de coopération Internationale en Recherche Agronomique pour le Développement (CIRAD), Kathmandu, Nepal; 5 Groningen Institute of Evolutionary Life Sciences, Groningen University, Groningen, Netherlands; 6 Departamento de Producción Agrícola y Animal, Universidad Autónoma Metropolitana Unidad Xochimilco, Mexico, Mexico; 7 International Maize and Wheat Improvement Center, Sustainable Intensification, Texcoco, Mexico; International Centre for Integrated Mountain Development (ICIMOD), Kathmandu, Nepal, NEPAL

## Abstract

Around 30% of global food is produced by smallholder farmers, yet they constitute the most food-insecure group. In Mexico, food self-sufficiency is declining. Rural policies in the country have stimulated the production of cash crops to the detriment of the traditional intercropping system, the *milpa*. Such a decline may have negative consequences for the food security of subsistence farmers. This study aimed to assess changes in nutritional self-sufficiency over the last 30 years and the role of *milpa* systems in food security for two communities in the highlands of Oaxaca, Mexico. The study used satellite images, censuses, and field data to estimate food production. Three cropping systems, monoculture of maize, monoculture of common bean, and the *milpa* were compared in terms of nutrients and vitamins produced. Furthermore, a household typology was developed for each community to contrast nutritional self-sufficiency levels between the different household types. Results showed that the *milpa* produced more volume of food per area compared to the other systems. The *milpa* also produced all the nutrients and vitamins (except for B12) required to feed at least 2 persons ha^-1^. Monocultures of maize lacked vitamins A, B9, B12, and C, and the common bean lacked vitamins A, B12, and C. While farmers recognized the importance of the *milpa*, they preferred monocultures due to the reduced labor demands of this system. Households that obtained most of their income from off-farm activities had the lowest nutritional self-sufficiency. Enhancing nutritional self-sufficiency through crop diversification has the potential to not only improve the nutrition of subsistence farmers, but also to enhance ecosystem service provision, promote biodiversity conservation and restoration, and improve resilience to climate change.

## Introduction

There is a long-standing debate on the role of food self-sufficiency as a strategy for food security [1,2]. Food self-sufficiency refers to the ability of a country, region, or household to reach their food requirements without the need for importing food [3]. Food security is met when people have food availability, access, use, and stability [3]. Recent estimations show that rural households with less than two hectares of land, supply 30–34% of global food [4], and yet, the majority of people suffering from food insecurity also live in rural areas [5]. This food insecurity can be a consequence of drought, conflicts, land pressure, and poverty [6]. Furthermore, food security is threatened by the shift from diverse cropping systems towards more simplified systems, such as monocropping [7]. In Mexico, nation-wide food self-sufficiency has been declining since the country began relying more on imported staple grains [8]. Indigenous people constitute the most food insecure group in the country [9] with 70% of the indigenous population living in poverty, as opposed to 40% of the non-indigenous population [10]. Indigenous groups oftentimes rely on diverse and traditional agricultural systems [11], which offer a range of crops that contribute to overall human nutrition [12]. Yet, no study has quantified the contribution of such traditional systems to the food security and nutritional self-sufficiency of the rural poor in Mexico. In this study, we show how food production and nutritional self-sufficiency (i.e. availability) have changed over time (i.e. stability), while also addressing the role of food diversity provided by traditional agricultural systems (i.e. use) in feeding the local population (i.e. access).

The North American Free Trade Agreement (NAFTA) of 1994 had several political and social implications for Mexico, most notably, decreasing the country’s food self-sufficiency and labor sovereignty (the ability of a nation to provide its citizens with living wages) and increasing out-migration (the process of moving from one place to another) [8]. To adjust to the NAFTA, Mexican policies incentivized households to move away from traditional food production systems and towards more economically productive crops [13]. Preibisch et al. [14] showed that although there was a strong disincentive to produce maize (*Zea mays* L.) after NAFTA, households continued to produce this staple crop. Novotny et al. (submitted) [15] showed similar results for the state of Oaxaca. They also found a decrease in crop areas associated with out-migration and an increase in off-farm income.

In Mexico, the traditional maize production systems called *milpa* provide the basis for rural households’ food supply. *Milpa* systems can be found throughout the Mexican territory [16], extending to South America [11]. In these systems, maize is usually inter-cropped with the common bean (*Phaseolus vulgaris* L.), and squash (*Cucurbita* spp.) [17]; with the crop species composition changing depending on the agroclimatic zone [11]. *Milpa* systems are important in affording food security because they increase household access to a diversity of food [16,18]. According to Mann [19], the diversity of crops found in the *milpa* is nutritionally and environmentally complementary. Although the *milpa* is a key component of the livelihood of rural families, only 6% of households can fully cover their needs from their own production [20]. In a study conducted in the Yucatan state, Leatherman et al. [21] related deficiencies in zinc and vitamins A and C to low *milpa* production. Nevertheless, the potential of the *milpa* to supply an adequate diet in terms of macro/micro-nutrients and vitamins and its role in nutritional self-sufficiency in rural areas, remains largely unexplored.

To assess food security, studies have addressed major staple crops such as maize, wheat, rice, and other grains, and their caloric and protein supply. While these crops are important sources of calories and protein, they are usually not sufficient to provide a balanced diet. This requires more nutrition-sensitive approaches [22,23]. Recent studies have called attention to aspects of food security such as diversity of food sources and the nutritional quality of products in terms of micro-nutrient and vitamin provision [24–26]. Remans et al. [7] adapted the functional diversity metric used in ecology to a nutritional functional diversity metric. This metric considers species composition as well as their nutritional contributions. Cassidy et al. [27] introduced an assessment of the number of people nourished per area in terms of calories and proteins, which DeFries et al. [28] later refined this to include more nutrients.

This study was carried out in the Mixteca Alta region, Oaxaca, where the political context has discouraged traditional systems, decreased interest in agricultural activities, and stimulated out-migration. We assessed how changes in the food production system associated with demographic changes have affected nutritional self-sufficiency in two communities in the state of Oaxaca, Mexico: Santa Catarina Tayata and San Cristóbal Amoltepec. Oaxaca is characterized by high migration levels, both nationally and internationally, and an increasing trend to pursue local off-farm activities as a livelihood strategy. These communities are representative of many others in Central America, in that the *milpa* still constitutes the basis of food production [11,29]. The objective of this study was to assess long-term changes in food demand and supply in a rural context and to analyze the role that the traditional agricultural systems play in local food security and nutritional self-sufficiency. The hypothesis is that *milpa* systems provide a balanced diet and that while demographic decline reduces the demand for food, it negatively affects *milpa* production due to reduced labor availability. To test our hypothesis, we investigated the following questions: 1) how has demographic decline caused by out-migration affected food security and nutritional self-sufficiency? 2) what are the nutritional benefits of the *milpa* compared to monocropping systems? and 3) what are the differences in nutritional self-sufficiency between household types? Answering these questions will inform the development of policies that support food security and the nutritional self-sufficiency of rural populations in conditions with net migration to cities.

## Methodology

This study, part of the ATTIC project (under grant agreement A4032.09.20), was approved by the Production Ecology & Resource Conservation graduate school at Wageningen University & Research (project number PE&RC 15101). Consent was obtained orally and the data was analyzed anonymously.

### Case study areas

**Fig 1:**
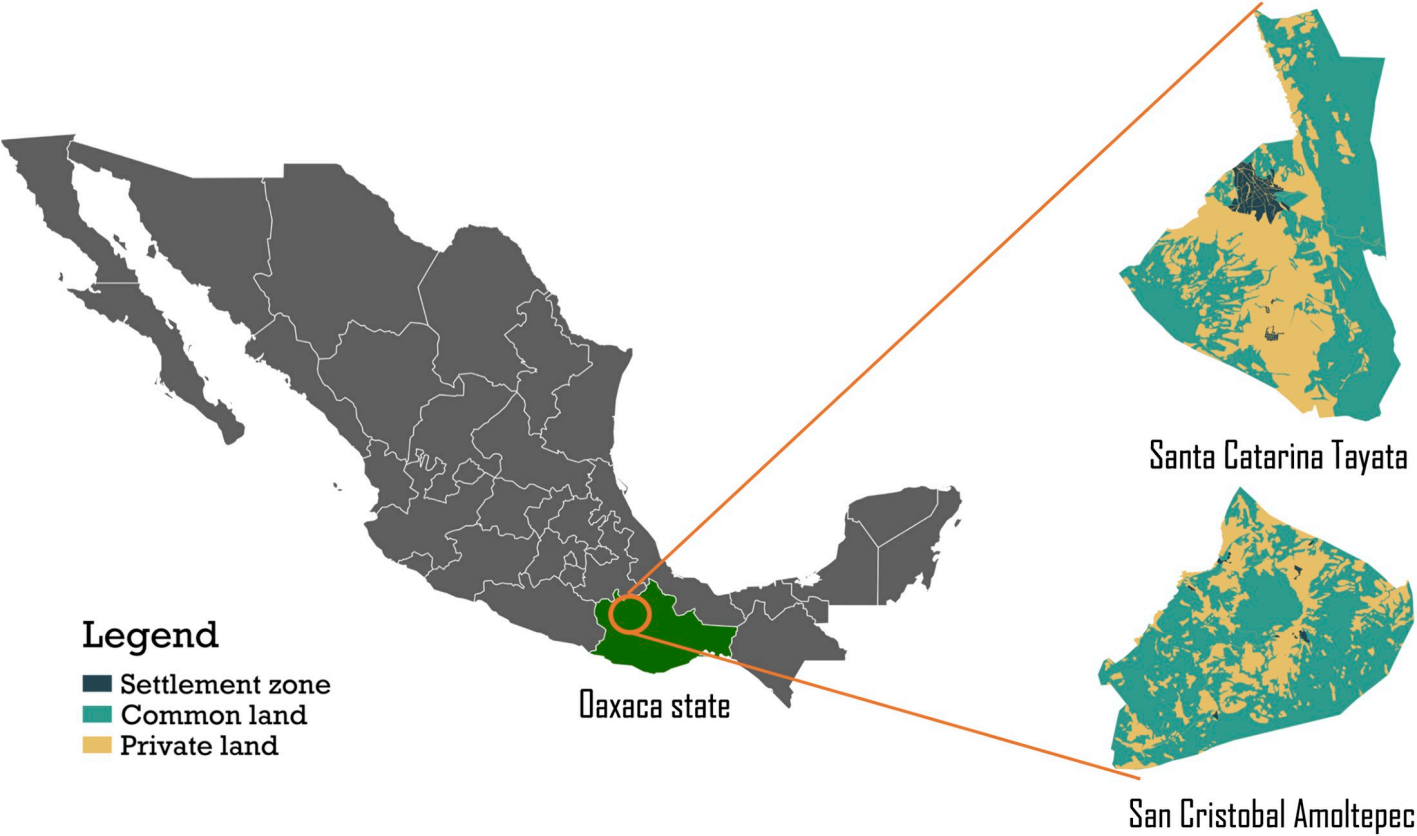
Location of the study areas of Santa Catarina Tayata and San Cristóbal Amoltepec within the state of Oaxaca (dark green in the map of Mexico).

Both case study municipalities are situated in the highlands of Oaxaca. Santa Catarina Tayata (SCT) (Fig 1) has an area of 37 square kilometers (km^2^) and is located at an elevation of between 2000 and 2500 meters (m) above sea level. The climate is sub-humid, with monthly average temperatures ranging from 16 to 18°C, and annual rainfall between 1000 and 1200 millimeters (mm). Land use in SCT is composed of settlements, private land, and communal land. Settlements are usually agglomerates of houses interspersed with cropland. Private land consists of cropland and fallow land. Communal land includes forests and grassland, both of which are managed by the municipality’s officials, who enforce rules for their management.

San Cristóbal Amoltepec (SCA) (Fig 1) is spread over 32 km^2^ at elevations of between 2100 and 2700 m. The climate is also sub-humid, with monthly average temperatures of 14 to18°C, slightly lower than SCT. Annual rainfall ranges from 800 to 1000 mm. Land use in SCA is distributed in the same way as in SCT.

The diversity in food produced varies across the area. Most simplified systems consist of maize and common bean grown as sole crops. *Milpa* systems in the area comprise maize intercropped with at least one of the following: common beans (*Phaseolus vulgaris*), fava bean (*Vicia faba*), and squash (*Cucurbita* spp.). Although sheep production can potentially provide animal protein and vitamin B12, it is usually used as a ‘savings account’, rather than for consumption. Common bean varieties differ depending on the system, exhibiting a climbing or creeping behavior when grown in the *milpa* or as a sole crop, respectively.

### Data sources

*Household survey*, *population censuses*, *agricultural censuses*, *and production survey*: A household survey was performed in December 2015 in SCT and in July 2016 in SCA. A total of 51 (25% of total) and 31 (10% of total) households were surveyed in SCT and SCA, respectively. The household survey included questions about family size, average crop production, migration, off-farm activities, and income sources. Demographic data at the municipal level was obtained from the population censuses of 1980, 1990, 1995, 2000, 2005, and 2010. Information on land size, cropping systems, crop yield, and animal production was gathered from the agricultural censuses of 1991 and 2007. To update the 2007 agricultural census, a survey was done between December 2018 and January 2019, and data on crop production, animal production, and labor requirements were collected. To estimate crop production, we visited 30 households in both SCT and SCA immediately after the harvest season. A total of 47 and 42 plots were considered for estimating crop production in SCT and SCA, respectively. Since farmers store their harvested maize and beans in 100 liter (L) tanks, 50 kilogram (kg) bags, or 4.5 kg cans, we counted these storage units as an estimate of total farm crop production. This method has proven to provide reliable results when performed soon after harvest [30]. We also counted the number of harvested squash fruits per farm, which we multiplied by an estimated average weight of 6.5 kg per fruit to obtain the total production in weight. To obtain crop yields in ton per hectare (t ha^-1^), we used Google Earth images with a shapefile containing all privately-owned plots from the *Registro Agrario Nacional* (2019b) [31]. With this image, the 30 farmers were asked to identify their crop fields, the crops grown on these, the proportion of a field allocated to each crop (in the case that more than one crop was grown), and the type of intercropping. To estimate animal production, we focused our questions on sheep husbandry since this was the most common type of animal husbandry in the area (although households also usually have between 5 and 10 chickens). Farmers were asked about the size of the flock, the fields used for grazing, and whether these fields were private property or belonged to the community. Based on the total grazing area and the number of sheep, we estimated the stocking rate (sheep ha^-1^). To calculate labor demands per cropping systems, farmers were asked how many people and days were required to produce on a specific plot.

Population sizes for 1991, 2007, and 2018, the years when agricultural census and production survey data were available, were estimated based on average growth rates derived from the population censuses. For instance, the average growth rate between 2005 and 2010 was used to estimate the population in 2007 based on the 2005 data. Similarly, the growth rate between 2010 and 2015 was used to project the population for 2018.

*Spatial data*: For up to 3 years before or after the agricultural censuses, Landsat satellite images were acquired and evaluated. Based on our understanding of landscape dynamics in the area [32], we assumed that landscape changes within the 3 years would have negligible influence on subsequent analyses. Image availability and cloud coverage limited the data to 1989, 2010, and 2017. The images selected had been taken at the end of the crop season, allowing robust differentiation of land uses. To facilitate the identification of the different land uses, we overlaid a high-resolution image from Google Earth (WorldView-2; resolution of 0.5 m) with a shapefile containing all fields registered in the municipalities [33].

### Calculations and analyses

#### Nutritional self-sufficiency at the municipal level

*Total cropping area at the municipal level*: To estimate the total crop production per municipality, a semi-supervised land use classification was performed to calculate the total cropland and grassland. This semi-supervised method consists of creating sample polygons based on visual interpretations of the different land uses. These polygons contain the average spectral signature that is used as input to train the classification algorithm and classify each pixel in the image [34]. To improve reflectance values we applied an atmospheric correction on the three Landsat images [35]. We used a high-resolution Google Earth image to select training samples for each aforementioned land use [36]. With the training samples, we applied a maximum likelihood algorithm, which resulted in a land use map for each of the 3 Landsat images [35,37].

*Actual crop production at the municipal level*: The production *P*_*hij*_ in the year *h* of crop *j* in municipality *i* in t ha^-1^ was calculated as:

$$P_{hij}\;=\;CA_{hi}*CP_{hij}*CY_{hij}$$ (1)

Where *CA* is the cropping area (ha, excluding fallow), *CP* is the proportion of the area occupied by a crop species, and *CY* is the crop yield (t ha^-1^).

*Actual sheep production at the municipal level*: Sheep production *SP* was derived from the average number of sheep per household and an animal-to-meat conversion rate of 15 kg per sheep. The conversion rate was obtained from interviews and is within the range mentioned for mutton production in extensive, grass-fed systems in Mexico [38].

*Actual total nutrient and vitamin production at the municipal level*: Since maize is mostly consumed in *tortilla* form, we transformed the maize kernel production to tortilla. Based on farmer information, 1 kg of air-dried maize kernels produced around 1.4 kg of fresh weight tortilla, as water and calcium oxide are added in the process. For each plant and animal food source identified in the survey, we derived the provision of calories, protein, calcium, iron, magnesium, phosphorus, and vitamins A, B2, B3, B6, B9, B12, and C, using the concentrations shown in Table 1.

**Table 1:** Nutritional characteristics of the major food sources produced in Santa Catarina Tayata and San Cristóbal Amoltepec, Oaxaca, Mexico.

Food source	Nutritional characteristics per 1 gram of food provided														Reference
	Energy (kcal^a^)	Protein (g^b^)	Calcium (mg^c^)	Iron (mg)	Magnesium (mg)	Phosphorus (mg)	Vit.A (μg^d^)	Vit. B2 (mg)	Vit. B3 (mg)	Vit.B6 (mg)	Vit. B9 (μg)	Vit. B12 (μg)	Vit. C (mg)	Zn (mg)	
Blue tortilla	0.0276	0.078	1.74	0.029	0.32	3.39	0.02	0.0017	0.039	0.001	0	0	0	0.0068	[39]
Yellow/white tortilla	0.0219	0.057	0.81	0.012	0.72	0.81	0	0.001	0.015	0.002	0	0	0	0.002	[39]
Common bean	0.0343	0.00227	1.34	0.071	0.6	4.15	0	0.0047	0.0209	0.0053	4.63	0	0.01	0.0255	[40]
Fava bean	0.0341	0.0026	1.03	0.067	1.92	4.21	0.03	0.0033	0.0283	0.0037	0	0	0.01	0.0314	[40]
Squash	0.003	0.00006	0.19	0.005	0.25	0.22	1.43	0.0004	0.005	0.0006	0.16	0	0.15	0.0032	[40]
Squash seeds	0.0612	0.0029	0.505	0.088	16.68	11.74^e^	0^e^	0.0015^e^	0.0443^e^	0.001^e^	0.57^e^	0^e^	0.065^e^	0.088^e^	[41]
Sheep meat	0.0267	0.0017	0.12	0.016	0.22	1.6	0	0.0022	0.061	0.0013	0	0.0239	0	0.0333	[40]

^a^kilocalorie.

^b^gram.

^c^milligram.

^d^microgram.

^e^Nutritional value for Cucurbita spp.; when not available for figleaf gourd (*Cucurbita ficifolia*).

The total nutrient or vitamin production *TNP*_*hli*_ (unit dependent on the nutrient and vitamin) in year *h* for nutrient *l* in municipality *i* was calculated as:

$$TNP_{hli}\;=\;\sum_{j\;=\;1}^{n}P_{hij}*NC_{lj}*1,000,000+\;{SP}_{hi}*NM_{lp}*1,000,000$$ (2)

Where *P*_*hij*_ is the production in year *h* of crop *j* in municipality *i*, *NC*_*lj*_ is the content of nutrient *l* in crop *j*, *SP*_*hi*_ is sheep production in year *h* and municipality *i*, and *NM*_*l*_ is the content of nutrient *l* in mutton. A conversion factor of 1,000,000 was used to transform 1 g per food source (Table 1) to t.

*Actual nutrient requirements and nutritional self-sufficiency at the municipal level*: Nutrient and vitamin requirements per person per year were derived from the Dietary Reference Intake (DRI) [42], applying the methodology used by de Ruiter et al. [43] and DeFries et al. [28]. The DRI was used for the age group of 31 to 50 (S1 Table), which is representative of the average age of the population. The requirements of pregnant or lactating women, as well as those of children were not considered in this study. The percentage of people fed *PF*_*lj*_ for a given nutrient or vitamin *l* in a municipality *j* was calculated as:

$$PF_{lj}\;=\;(\frac{\frac{TNP_{li}}{D_{l}}}{POP_{j}})*100$$ (3)

Where *D*_*l*_ is the annual demand for nutrient or vitamin *l* (derived from 37) and *POP*_*j*_ is the total population in municipality *j*.

*Average nutrient requirements and nutritional self-sufficiency at the municipal level*: Apart from calculating actual nutritional self-sufficiency, we also calculated average nutritional self-sufficiency to minimize the effects of yield variation between cropping cycles. Average yields were reported by farmers during the survey in 2018. We repeated the steps above using the average crop yield to calculate average nutritional self-sufficiency.

#### Cropping systems comparison

*Nutrient and vitamin production per system*: The system nutrient or vitamin production SNP_jlm_ (unit dependent on the nutrient or vitamin) for nutrient or vitamin *l* produced in system *m* (i.e. *milpa*, sole maize, and sole bean) and in municipality *j* was calculated as:

$$SNP_{jlm}\;=\;\sum_{j\;=\;1}^{n}CY_{ij}*NC_{lj}*1,000,000$$ (4)

*Labor*: Labor efficiency *L*_*io*_ of cropping system *o* in municipality *i* in kilogram per hour per hectare (kg h^-1^ ha^-1^) was calculated as:

$$L_{io}\;=\;\frac{\frac{\sum_{j\;=\;1}^{n}CY_{ij}}{1000}}{W_{io}*6}$$ (5)

Where *W* is the total number of working days required in a cycle, 1000 is the t to kg conversion factor, and 6 is the conversion factor from working day to hours.

#### Nutritional self-sufficiency at the household level

*Household typology*: A principal component analysis followed by a hierarchical cluster analysis was performed to classify households into types [44–46]. The following variables from the household survey were used in the analysis: family size, number of household members who migrated, income source (e.g. agriculture, off-farm, remittances, and subsidies), cropping area, number of crop species, and tropical livestock units (TLU) owned. After grouping the households into types, the nutritional self-sufficiency per type was calculated and compared.

*Nutritional self-sufficiency at the household level*: Nutritional self-sufficiency at the household level was calculated using Eq 3 but considering the *TNP* at the household level and the total number of inhabitants per household.

*Cropping systems comparison*: Based on our survey, average crop yields for the different cropping systems *m* were compared by the non-parametric Kruskal-Wallis test, using the PMCMR (version 2016-01-06) package in R (version 3.6.1).

## Results

### Population and food production dynamics

**Fig 2:**
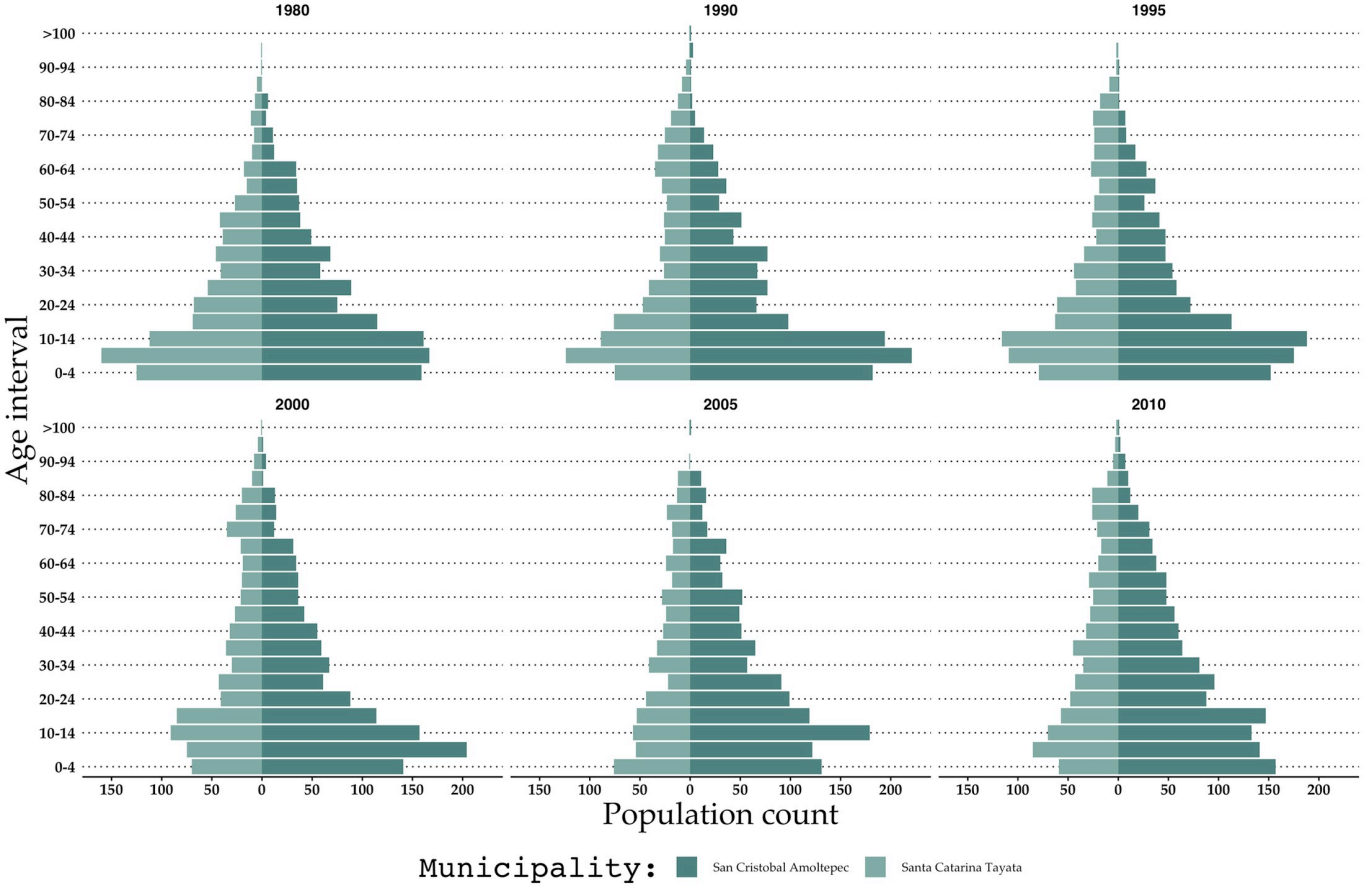
Age pyramids for Santa Catarina Tayata and San Cristóbal Amoltepec for the years 1980, 1990, 1995, 2000, 2005 and 2010 [48–53].

Between 1980 and 2015 the population in SCT declined from 864 to 679. In SCA, the population remained close to 1200 persons until 2010. From 2010 to 2015, however, the population declined from 1271 to 1004 (INEGI 2015, 2010, 1990) [47–49]. In SCT, there were 400 potential workers (aged between 16 and 60 years) in 1980 and 342 in 2010, with the lowest value of 290 recorded in 2005 (Fig 2). The portion of the population above the age of 60 increased from 33% to 94%. The increase of senior inhabitants may be the result of higher life-expectancy and an older population returning to their municipalities for retirement (a common phenomenon in SCT). The age pyramid thus shows an out-migration of people of working age. In SCA, the age structure was characterized by a large base of young people and a narrow tip of older people.

Between 1989 and 2017 the cropping area decreased by 88 ha in SCT and by 113 ha in SCA. Despite a reduction in cropping activities, SCT showed higher maize yields and produced a larger volume of food with less area in 2018 compared to 1991 and 2007 (Table 2). In comparison with SCT, crop yields in SCA were lower for every year assessed.

**Table 2:** Food production in Santa Catarina Tayata (SCT) and San Cristóbal Amoltepec (SCA) for the years 1991, 2007 (agricultural census), and 2018 (survey).

Community	Year	Cropland	Grassland	Blue maize		White maize		Yellow maize		Common bean		Fava bean		Squash flesh		Squash seed		Sheep	
		Ha	ha	ha	t^a^	ha	t^a^	ha	t^a^	ha	t^b^	ha	t^b^	ha	t^c^	ha	t^c^	Heads	Meat (t)
SCT	1991	546	897	31	12.9	282	118.4	125	52.6	273	57.3	77	0.9	45	34.5	45	2.0	960	14
	2007	542	581	23	16.6	213	153.4	95	68.1	223	49.2	76	0.9	45	34.2	45	2.0	1253	19
	2018	458	658	31	26.2	288	241.8	128	107.4	257	38.6	65	0.8	38	29.0	38	1.7	1920	29
SCA	1991	579	1161	106	39.3	209	77.2	219	80.9	91	8.9	41	1.0	66	9.2	66	0.5	526	8
	2007	417	659	46	23.2	91	45.6	96	47.8	101	19.2	30	0.7	47	6.2	47	0.4	716	11
	2018	466	759	78	26.7	154	52.5	162	55.0	337	18.6	33	0.8	53	7.4	53	0.4	2450	37

^a^kernel production in ton.

^b^dry bean production in ton.

^c^edible part in ton.

**Fig 3:**
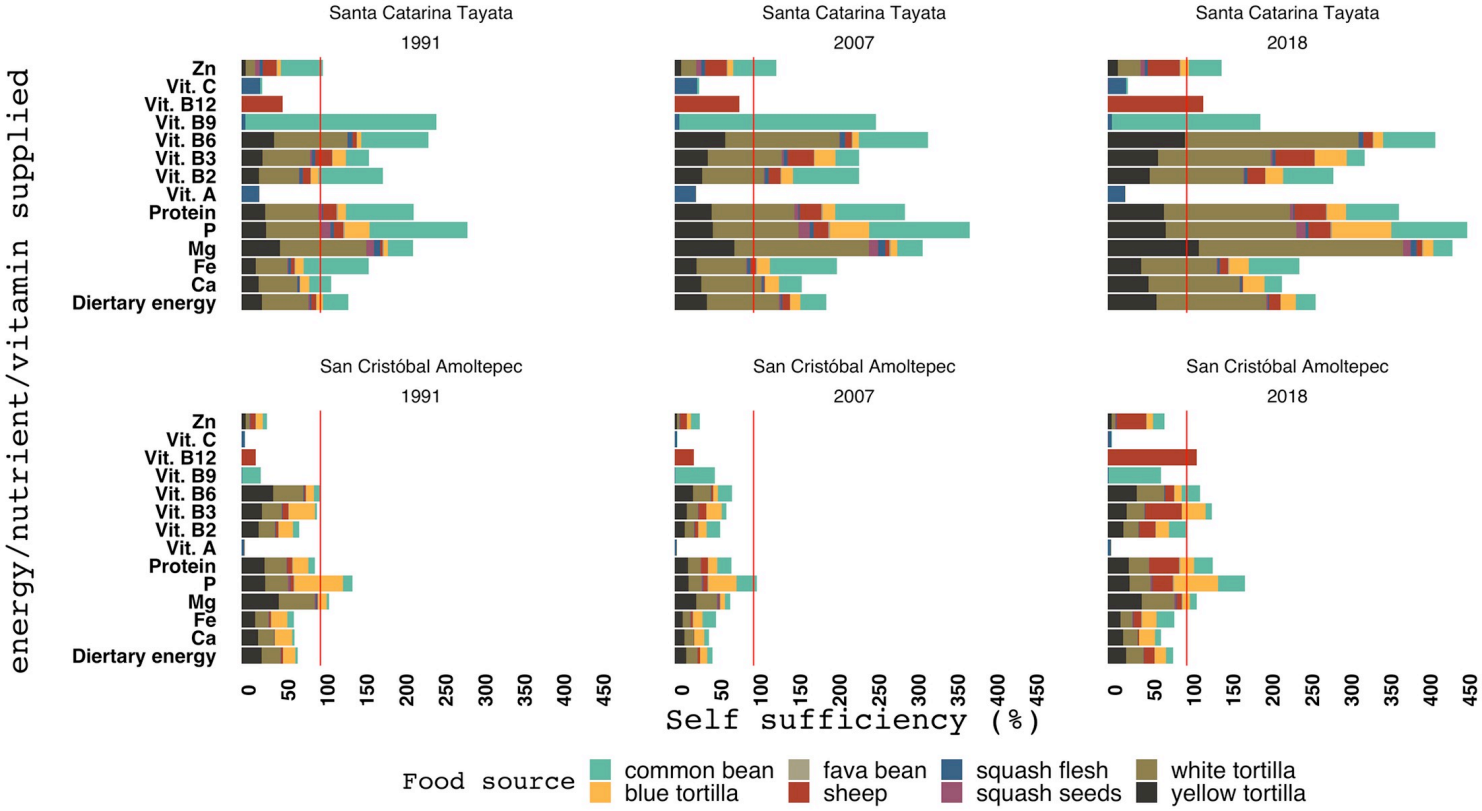
Relative supply of dietary energy (kcal), protein (g), calcium (mg), iron (mg), magnesium (mg), phosphorus (mg), vitamin A (μg), B2 (mg), B3 (mg), B6 (mg), B9 (μg), B12 (μg), C (mg) by the different food sources in Santa Catarina Tayata (panels on the left) and San Cristóbal Amoltepec (panels on the right), in 1991, 2007 and 2018. The vertical line represents the population demand reference, set at 100%.

The variation in crop yields, total cropping area, and population size across the years resulted in different levels of nutritional self-sufficiency (Fig 3). Overall, maize supplied the majority of nutrients and vitamins, but it lacked Zn and vitamins A, C, B9, B12, and C. SCT sustained food production to meet its population demands for nutrients and vitamins, with the exception of vitamins A, C, and B12. SCA did not reach self-sufficiency for several nutrients and vitamins. With a population of 1212 and low crop yields in 2007, SCA only reached self-sufficiency in phosphorous.

**Fig 4:**
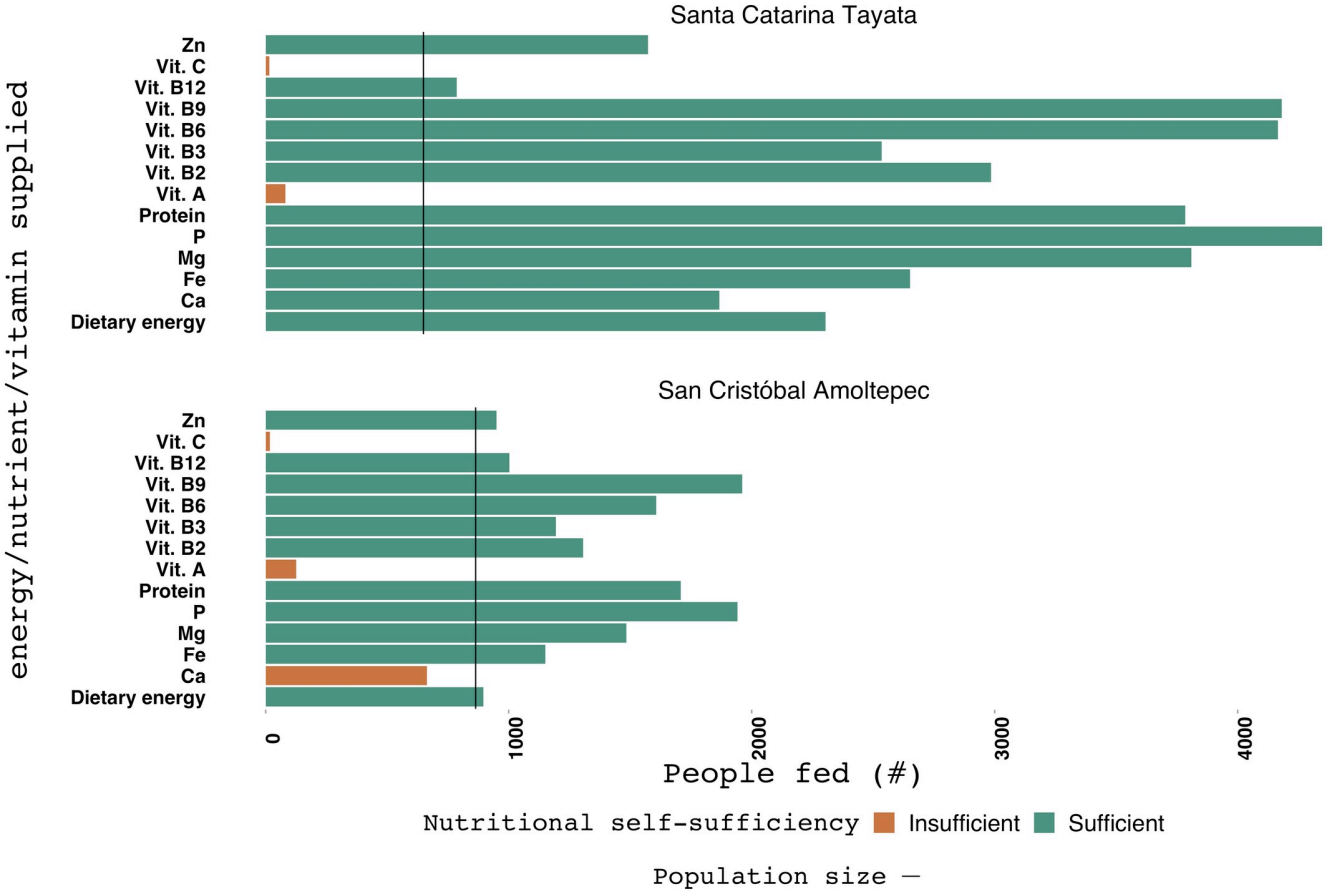
Estimated nutritional self-sufficiency for Santa Catarita Tayata and San Cristóbal Amoltepec based on average crop yields and animal production levels.

Farmers in SCA reported that yields were below average in 2018. With average crop yields, SCA was shown to be self-sufficient for most nutrients and vitamins, except for Ca and vitamins A and C (Fig 4). Nevertheless, even with average crop yields there is little surplus of Zn and dietary energy, which indicates nutritional self-sufficiency sensitivity to changes in crop yields and population size in SCA.

### The role of the traditional intercropping system in nutrition

**Fig 5:**
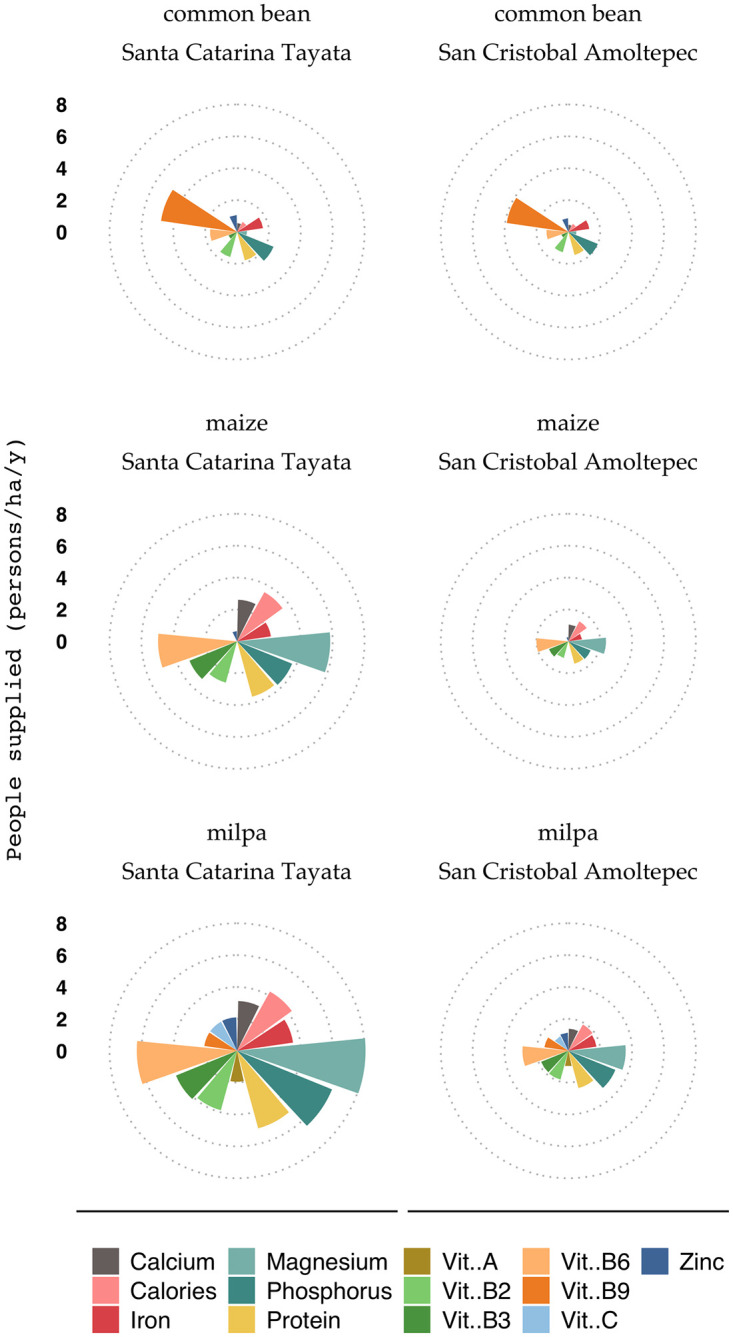
Provision of nutrients and vitamins by maize and the common bean as sole crops and by the milpa, consisting of community-specific mixtures of maize, common bean, fava bean, and squash expressed in persons per ha per year. Each dotted circle represents 2 persons per ha per year. Based on yield data of 2017/2018 for Santa Catarina Tayata and San Cristóbal Amoltepec.

The *milpa* was the least common cropping system in 2018. It occupied around 5% and 10% of the total cropland of SCT and SCA, respectively. *Milpa* systems provided more persons per hectare with a complete set of nutrients and vitamins than sole crops of maize or common bean (Fig 5). Except for zinc and vitamins A, C, B9, and B12, *milpa* systems provided complete nutrition sufficiency for up to 3 persons ha^-1^ depending on crop yields and crop composition, while sole crops of maize and common bean provided enough to feed up to 2 and 0.5 persons ha^-1^, respectively.

As expected, all cropping systems performed better in SCT, as crop yields in the municipality were consistently higher than in SCA (Table 2). Maize plant density and yield in the *milpa* and as a sole crop were not statistically different in either community. Common bean yield in the *milpa* was significantly lower by a factor 3 than when grown as a sole crop (Kruskal-Wallis, *P = 0*.*025*). This was largely explained by the difference in plant density. In the *milpa*, maize constitutes the main crop while other species are normally added to the system in varying densities. Common bean is added as second crop at a plant density of 40,000 plants ha^-1^ compared to 100,000 plants ha^-1^ when grown as a sole crop. Farmers preferred to keep a lower common bean density in the *milpa* to avoid competition and consequently decrease of maize yield. Since fava bean and squash were only produced in the *milpa*, we could not compare their yields to sole crops. One hectare of traditional *milpa* provided enough nutrients and vitamins to feed at least two people per year in SCT and one in SCA when including zinc, vitamin A, C, B9, and B12.

Comparing labor efficiency of the different cropping systems for each municipality, SCT produced more food per hour of labor than SCA (Table 3). In each municipality, maize systems produced the most food per hour of labor, with 5.0 kg h^-1^ ha^-1^ in SCT and 2.0 kg h^-1^ ha^-1^ in SCA. Bean had the lowest performance with 0.8 kg h^-1^ and 0.6 kg h^-1^ for SCT and SCA, respectively. When compared to maize, the milpa produced less food per hour worked. The difference in labor efficiency between maize and the milpa was 1.7 kg h^-1^ ha-1 in SCT and 0.2 kg h^-1^ ha^-1^ in SCA.

**Table 3:** Labor efficiency and nutritional self-sufficiency for common bean, maize, and the milpa in Santa Catarina Tayata (SCT) and San Cristóbal Cristobal Amoltepec (SCA).

Cropping system	Labor efficiency (kg h^-1^ ha^-1^)		People fed on calory produced (persons fed ha^-1^)	
	SCT	SCA	SCT	SCA
Common bean	0.79	0.6	0.7	0.6
Maize	5.02	2.0	3.5	1.4
Milpa	3.32	1.8	4.2	1.9

### Nutritional self-sufficiency per household type

**Fig 6:**
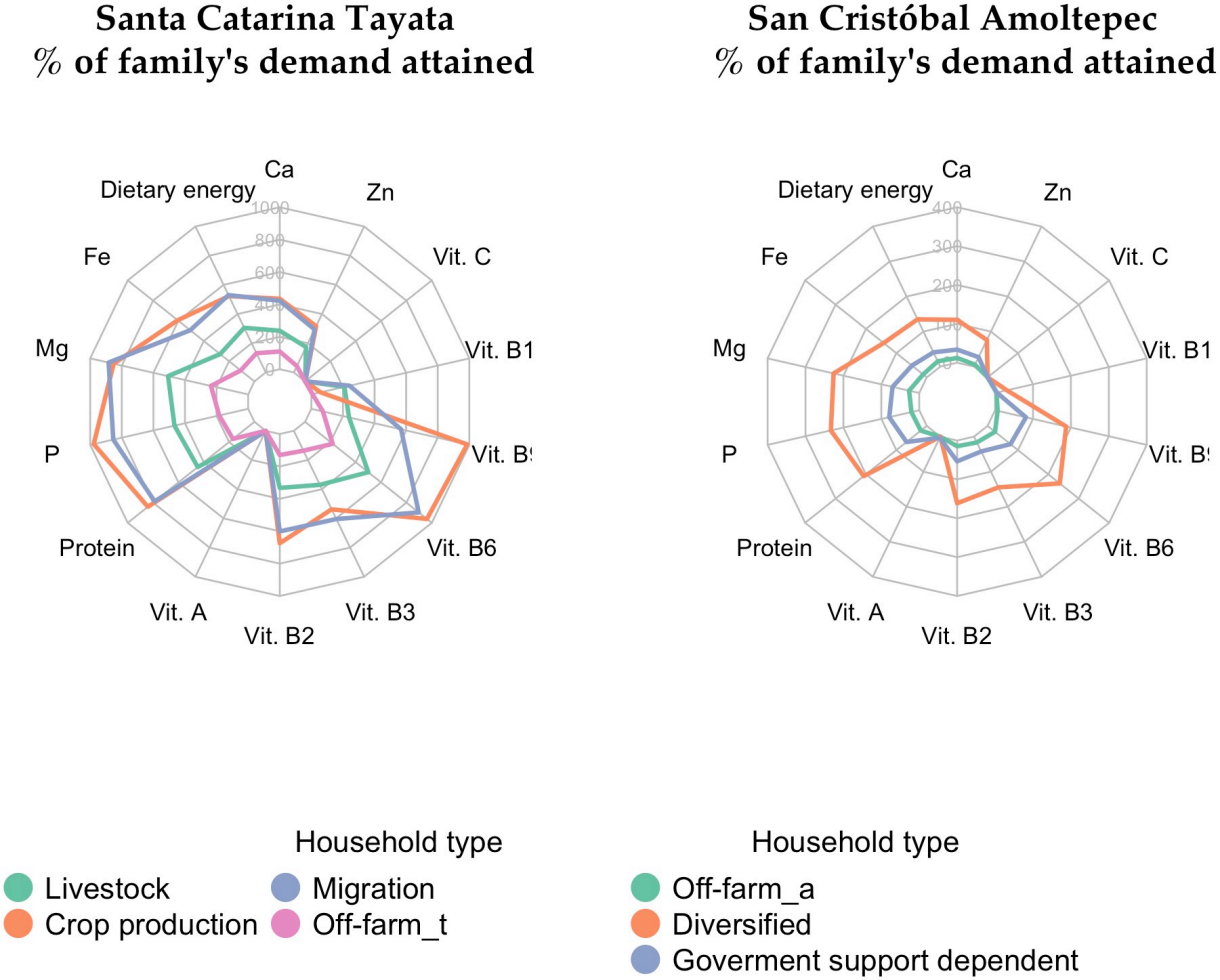
Number of times a family is provided with a given nutrient or vitamin. Different colors represent different household types.

A total of four and three household types were identified in SCT and SCA, respectively (Table 4). None of the household types produced sufficient amounts of vitamins A and C to meet their demands (Fig 6). Excluding these vitamins, the livestock and migration household types were self-sufficient. The relatively high level of self-sufficiency of the migration type was attributed to small households of an average of two persons (S6 Table). The crop production type had similar levels of self-sufficiency to the migration type. Nevertheless, low animal production levels in the former group were reflected in an insufficient supply of vitamin B12. Despite having the lowest nutritional self-sufficiency, the off-farm type in SCT produced enough nutrients and vitamins to meet the household demand, except for vitamins A, C, and B12.

**Table 4:** Household types in Santa Catarina Tayata and San Cristóbal Amoltepec.

Municipality	Household type	Main characteristics
Santa Catarina Tayata	Livestock	Mid-size family (4 inhabitants), low migration of family members (0 to 1 person), and 9 months of maize and bean self-sufficiency. Low number of cultivated crops (only maize and bean), focus on livestock production (TLU = 4.8) and medium contribution of animal production to the total income (34%).
	Crop production	Mid-size family, low migration of family members (0 to 1 person), and 11 to 12 months of maize and bean self-sufficiency. Medium animal production (TLU = 2.0) and medium-high number of cultivated crops (3 to 4 species). Medium contribution of cropping activities to the total income (20%).
	Migration	Mid-size family, medium-high migration of family members (2 to 6 persons), and 11 to 12 months of maize and bean self-sufficiency. Medium animal production (TLU = 2.0) and medium-high number of cultivated crops (3 to 4 species). Medium contribution of remittances to the total income (20%).
	Off-farm	Large-size family (6 inhabitants), low migration of family members (0 to 1 person), and 9 months of maize and bean self-sufficiency. Low animal production (TLU = 1) and a low number of cultivated species (2). Very high contribution of off-farm activities to the total income (90%).
San Cristóbal Amoltepec	Off-farm	Mid-size family, low migration of family members (0 to 1 person), and 10 months of maize and bean self-sufficiency. Low animal production, low number of cultivated species (1 to 2), high contribution of off-farm activities to the total income (60%).
	Diversified	Small-size family (3 inhabitants), low-medium migration of family members (0 to 2 persons), and 9 months of maize and bean self-sufficiency. Low animal production and medium number of cultivated species (3). Medium contribution of off-farm activities (45%) and government support to the total income (30%).
	Government support-dependent	Medium-size family, low migration of family members (0 to 1 person), and 9 months of maize and bean self-sufficiency. Low animal production and medium-high number of cultivated species. High contribution of government support to the total income (70%).

In SCA, the diversified type was the only household type that achieved self-sufficiency for most nutrients and vitamins. The government support dependent type and the off-farm type did not achieve self-sufficiency for any nutritional element considered.

## Discussion

Although SCT had a smaller cropping area, crop yields were higher and the population size smaller, resulting in greater surpluses of calories and protein. In SCA, a demographic decline from 1991 led to a decrease in the demand for food. Nevertheless, the low crop yields precluded achieving nutritional self-sufficiency for more than half of the nutritional elements assessed. SCA achieved nutritional self-sufficiency for all nutritional elements, except for dietary energy and vitamins A and C. Maize production provided greater quantities of most nutrients and vitamins in both SCT and SCA. The *milpa* provided a complete set of dietary energy, protein, vitamins, and micronutrients. Due to higher productivity levels in SCT, the *milpa* system fed up to 2 persons ha^-1^ y^-1^ with a deficiency of vitamins A, B9, and C, and zinc, and up to 4 and 5 persons ha^-1^ y^-1^ when dietary energy and protein were considered, respectively. The *milpa* yield for SCA was around half that of SCT. Monocultures of common bean and maize were deficient in several micronutrients and vitamins. Sole maize had a clear advantage over other cropping systems when considering labor efficiency, which explained why households tended to grow sole maize instead of the *milpa*. Although the common bean was the cropping system that produced the least volume per hour worked, households preferred to consume the creeping variety (grown as a sole crop) over the climbing variety (grown in the *milpa* system). The level of nutritional self-sufficiency depended on the household type. Overall, households that dedicated more time to off-farm activities had the lowest nutritional self-sufficiency. While every household type in SCT was able to fulfill nutrient and vitamin needs (except vitamins A and C), only the diversified household type in SCA provided sufficient amounts of nutrients for the family.

Out-migration from the state of Oaxaca intensified in the late 1960s and led to demographic declines in various communities [54]. Low food security can trigger migration processes, and migration, in turn, can support agricultural activities through remittances [55,56] or increase households’ capacities to buy food [57–59]. At the same time, the departure of a family member reduces labor availability, decreases pressure on resources, and increases food availability [60]. These arguments show that migration does not necessarily improve food security through investments in agriculture and, in some cases, can harm production systems because of reduced labor availability. As such, food sovereignty, which can be defined as, “the right of people to healthy and culturally appropriate food produced through ecologically sound and sustainable methods, and their right to define their food and agriculture systems” [61], can be negatively affected. Sunam and Adhikari [62] used both the principles of food security and food sovereignty to explain the impacts of migration on food production and consumption in Nepal. Our data show similar connections between migration, food security, and food sovereignty. Results showed that the *milpa* has lower labor self-sufficiency when compared to sole maize. During the survey, farmers in the area showed that they understood that the *milpa* could produce more food volume but they argued that it required a high degree of labor (Table 4). This reflected other studies that have also related lack of interest in *milpa* systems to its relatively high labor demand [63,64].

In recent years, studies of the dietary contribution of crops have shifted from a focus on energy and protein to the assessment of a set of nutritional values [7,23,28,43,65,66]. The *milpa* had a clear advantage over monocropping systems, especially concerning vitamins A and C, and Zn. Leatherman et al. [21] found a relationship between low *milpa* production and deficiencies of vitamins A and C, and Zn in Yucatán. Also in Yucatán, Calix de Dios et al. [67] showed that the *milpa* provided a little over six months of food self-sufficiency to the majority of households. Other studies have shown the advantage of polycropping systems for improving nutrition [7,68,69]. Our results showed that, in the municipality with lower crop yields, the *milpa* was particularly important since it provided higher crop diversity and produced more food per area when compared to monocrops of maize or bean.

To bring *milpa* production back into farmers’ systems for improving food security and nutritional self-sufficiency, efforts should target reducing required labor (or producing more with similar amounts of labor). Flores-Sánchez et al. [70] showed that *milpa* production could be improved through better plant nutrition. Reyna-Ramírez et al. [71] found higher *milpa* production in organic systems. Since weeding is the most laborious activity in the *milpa*, Parsons et al. [72] recommended using herbicides mid-season to avoid harming companion crops. Recommendations based on herbicide application should be considered prudently, as herbicides damage edible weeds (called *quelites*) that are usually also harvested in *milpa* systems [73]. Mulching has been recommended as a weed-suppression measure for several crops, and it could offer an alternative to hand weeding [74–77]. However, mulching croplands with residues would compete with fodder for animal nutrition. Finally, designing or adapting tools that would facilitate weeding in the *milpa* could facilitate weeding management and encourage farmers to use the system.

Household engagement in off-farm activities and migration highlighted the differences in nutritional self-sufficiency between household types. In families marked by migration, the small household size resulted in lower demands for food. Consequently, these families produced sufficient food to meet their needs in SCT. In the Mexican context, the lack of available labor caused by migration is usually counteracted by hiring labor through remittance money, as shown by Cano et al. [78] and Barrientos and Magaña [79]. Households that dedicated more time to off-farm activities had lower food production and therefore lower nutritional self-sufficiency. Contrastingly, in an econometric study at a national level in China, Yang et al. [80], showed that neither migration nor off-farm income negatively affected production. Given the low crop yields in SCA, households struggled to reach their nutritional self-sufficiency. The diversified household type was the only group that managed to maintain their nutritional self-sufficiency. Low crop yields and self-sufficiency for smallholder farmers are widespread problems in Mexico, which is reflected in the country’s high importation of staple foods such as maize and beans [81].

While this study focused on two case study locations, the use of the *milpa* is decreasing across Mexico, and we showed how this affects communities’ food security, food sovereignty, and nutritional self-sufficiency. The role of the *milpa* for food security is recognized [18,67,74,82], but this is, according to our knowledge, the first report of the nutritional value of a *milpa* system in terms of how many persons it can feed per area. The results will vary among locations, given the diversity of *milpa* systems [11]. The contribution of diversified cropping systems to greater nutritional self-sufficiency found in this study, however, may be applicable for other food-insecure areas in Latin America, Africa, and Asia [1,1,83–85]. Furthermore, promoting nutritional self-sufficiency through crop diversification can contribute to several political agendas in the field of biodiversity conservation, provision of ecosystem services, and climate change resistance and resilience. For instance, Gurr et al. [86] showed that using crop diversification as an ecological intensification technique contributed to natural pest control in rice fields in China, Thailand, and Vietnam, thereby reducing 70% of the pesticide application and increasing economic gains by 7.5%. Kremen and Miles [87] inspected 12 studies to contrast monocropping systems with diversified system. They concluded that diversified systems offered clear benefits regarding pollination, carbon sequestration, soil quality, and were more resilient to climate change.

This study had some limitations. Given the number of surveyed households and the number of plots considered, the performance of different cropping systems is unlikely to be representative of larger regions in Oaxaca. Nevertheless the benefits of the *milpa* may reflect those of other communities similar to SCA, especially when considering that Oaxaca is the third poorest state in Mexico [10] and had the sixth lowest maize yield in 2019 [88]. Each municipality produced adequate quantities of vitamin B12 from meat. Animals, however, usually act as safety-nets for households to deal with financial stresses, rather than as a food source. Therefore, vitamin B12 provision is likely to be less than what we calculated. Future research could test blood samples for deficiencies, along with dietary recall that includes the provenance of the food eaten [7,89]. Although we showed that food production in the area was not providing sufficient vitamin C, this does not necessarily mean that the population suffered from vitamin C deficiency. During our surveys, we observed that many households had citrus trees in their yard. Such minor food sources are not usually considered in the census data, making it harder to obtain a full panorama of food security and nutritional self-sufficiency. We focused on the nutritional supply and the population demand on an annual basis. This does not reflect food availability and consumption year-round, as several studies have shown that households usually face shortages in the months before the harvest season [67,90–93]. Obtaining nutritional values for the different locally grown crops proved to be difficult. Even though Mexico is a hot spot for genetic diversity of maize and the common bean, very few studies are available that reveal the diversity in terms of nutritional value, which may be further affected by the production method and location [94,95]. Finally, in our analyses, we used the average population age for the dietary intake and did not consider children or pregnant women. The inclusion of these groups would provide a more nuanced perspective, as these categories are known to be particularly sensitive to nutritional deficiencies.

## Conclusions

At the municipal level, the combination of a large population and low crop yields poses a threat to nutritional self-sufficiency. The preference for a few monocultures (e.g. maize and beans) reduces the diversity of food produced, and may lead to a shortage of micronutrients and vitamins. As such, traditional cropping systems with a diverse species composition are recognized as being important for improving food security and the nutritional quality of diets. In the cases of the municipalities under study, the lack of available labor shifted farmers’ preferences towards monocropping systems. Consequently, the production of diversified food was insufficient to meet the population’s demand for a nutritiously balanced diet. Failing to achieve nutritional self-sufficiency can be particularly problematic for the impoverished and fragile rural communities. In such places, sustained self-sufficiency would provide a safety net for extreme conditions. Enhancing diverse production should thus address two aspects: stimulate households to produce diversified cropping systems, and increase crop yields. Given the low access to inputs in marginalized areas, agricultural development should focus on stimulating practices that maximize natural resources used to increase crop yields (e.g. ecological intensification). By promoting crop diversification rooted in ecological principles, political agendas could address problems regarding biodiversity, low ecosystem services provision, and poor resilience to climate change.

## Supporting information

S1 Table Dietary Reference Intakes (DRIs). (PDF)

S2 Table Population count per age group. (PDF)

S3 Table Percent of the population served with nutrients and vitamins per food source. (PDF)

S4 Table Number of persons served with nutrients and vitamins under average crop yield. (PDF)

S5 Table Number of persons served with nutrients and vitamins per hectare per cropping system. (PDF)

S6 Table Descriptive statistics of household types. (PDF)
