# Supplementary material for: The importance of the traditional milpa in food security and nutritional self-sufficiency in the highlands of Oaxaca, Mexico
Source: PLoS One. 2021 Feb 19;16(2):e0246281. doi: 10.1371/journal.pone.0246281 (PMC7894926; doi:10.1371/journal.pone.0246281)
Supplement: S1 Table — (PDF) [file pone.0246281.s001.pdf]

Dietary Reference Intakes (DRIs): Recommended Dietary Allowances and Adequate Intakes per Life Stage Group												
**Life Stage Group**	**Protein[b] (g/d)**	**Calcium (mg/d)**	**Iron (mg/d)**	**Magnesium (mg/d)**	**Phosphorus (mg/d)**	**Zinc (mg/d)**	**Potassium (g/d)**	**Vitamin A (µg/d)[a]**	**Vitamin C (mg/d)**	**Niacin (mg/d)[e]**	**Vitamin B_6 (mg/d)**	**Folate (µg/d)[f]**
Infants												
0–6 mo	9.1*	200*	0.27*	30*	100*	2*	0.4*	400*	40*	2*	0.1*	65*
6–12 mo	**11.0**	260*	**11**	75*	275*	**3**	0.7*	500*	50*	4*	0.3*	80*
Children												
1–3 y	**13**	**700**	**7**	**80**	**460**	**3**	3.0*	**300**	**15**	**6**	**0.5**	**150**
4–8 y	**19**	**1,000**	**10**	**130**	**500**	**5**	3.8*	**400**	**25**	**8**	**0.6**	**200**
Males												
9–13 y	**34**	**1,300**	**8**	**240**	**1,250**	**8**	4.5*	**600**	**45**	**12**	**1.0**	**300**
14–18 y	**52**	**1,300**	**11**	**410**	**1,250**	**11**	4.7*	**900**	**75**	**16**	**1.3**	**400**
19–30 y	**56**	**1,000**	**8**	**400**	**700**	**11**	4.7*	**900**	**90**	**16**	**1.3**	**400**
31–50 y	**56**	**1,000**	**8**	**420**	**700**	**11**	4.7*	**900**	**90**	**16**	**1.3**	**400**
51–70 y	**56**	**1,000**	**8**	**420**	**700**	**11**	4.7*	**900**	**90**	**16**	**1.7**	**400**
> 70 y	**56**	**1,200**	**8**	**420**	**700**	**11**	4.7*	**900**	**90**	**16**	**1.7**	**400**
Females												
9–13 y	**34**	**1,300**	**8**	**240**	**1,250**	**8**	4.5*	**600**	**45**	**12**	**1.0**	**300**
14–18 y	**46**	**1,300**	**15**	**360**	**1,250**	**9**	4.7*	**700**	**65**	**14**	**1.2**	**400**[i]
19–30 y	**46**	**1,000**	**18**	**310**	**700**	**8**	4.7*	**700**	**75**	**14**	**1.3**	**400**[i]
31–50 y	**46**	**1,000**	**18**	**320**	**700**	**8**	4.7*	**700**	**75**	**14**	**1.3**	**400**[i]
51–70 y	**46**	**1,200**	**8**	**320**	**700**	**8**	4.7*	**700**	**75**	**14**	**1.5**	**400**
> 70 y	**46**	**1,200**	**8**	**320**	**700**	**8**	4.7*	**700**	**75**	**14**	**1.5**	**400**
Pregnancy												
14–18 y	**71**	**1,300**	**27**	**400**	**1,250**	**12**	4.7*	**750**	**80**	**18**	**1.9**	**600**[j]
19–30 y	**71**	**1,000**	**27**	**350**	**700**	**11**	4.7*	**770**	**85**	**18**	**1.9**	**600**[j]
31–50 y	**71**	**1,000**	**27**	**360**	**700**	**11**	4.7*	**770**	**85**	**18**	**1.9**	**600**[j]
Lactation												
14–18	**71**	**1,300**	**10**	**360**	**1,250**	**13**	5.1*	**1,200**	**115**	**17**	**2.0**	**500**
19–30 y	**71**	**1,000**	**9**	**310**	**700**	**12**	5.1*	**1,300**	**120**	**17**	**2.0**	**500**
31–50 y	**71**	**1,000**	**9**	**320**	**700**	**12**	5.1*	**1,300**	**120**	**17**	**2.0**	**500**
Values extracted from the Dietary Reference Intake tables found on https://ods.od.nih.gov/HealthInformation/Dietary_Reference_Intakes.aspx												

Vitamin B_{12} (µg/d)
0.4*
0.5*
**0.9**
**1.2**
**1.8**
**2.4**
**2.4**
**2.4**
**2.4**[h]
**2.4**[h]
**1.8**
**2.4**
**2.4**
**2.4**
**2.4**[h]
**2.4**[h]
**2.6**
**2.6**
**2.6**
**2.8**
**2.8**
**2.8**
