# Supplementary material for: The importance of the traditional milpa in food security and nutritional self-sufficiency in the highlands of Oaxaca, Mexico
Source: PLoS One. 2021 Feb 19;16(2):e0246281. doi: 10.1371/journal.pone.0246281 (PMC7894926; doi:10.1371/journal.pone.0246281)
Supplement: S2 Table — (PDF) [file pone.0246281.s002.pdf]

	Age interval	Municipality	Year	Number of people
1	0_4	San Cristobal Amoltepec	1980	159
2	5_9	San Cristobal Amoltepec	1980	167
3	10_14	San Cristobal Amoltepec	1980	161
4	15_19	San Cristobal Amoltepec	1980	115
5	20_24	San Cristobal Amoltepec	1980	75
6	25_29	San Cristobal Amoltepec	1980	89
7	30_34	San Cristobal Amoltepec	1980	58
8	35_39	San Cristobal Amoltepec	1980	68
9	40_44	San Cristobal Amoltepec	1980	49
10	45_49	San Cristobal Amoltepec	1980	38
11	50_54	San Cristobal Amoltepec	1980	37
12	55_59	San Cristobal Amoltepec	1980	35
13	60_64	San Cristobal Amoltepec	1980	34
14	65_69	San Cristobal Amoltepec	1980	12
15	70_74	San Cristobal Amoltepec	1980	11
16	75_79	San Cristobal Amoltepec	1980	4
17	80_84	San Cristobal Amoltepec	1980	6
18	85_89	San Cristobal Amoltepec	1980	0
19	90_94	San Cristobal Amoltepec	1980	0
20	95_99	San Cristobal Amoltepec	1980	0
21	>100	San Cristobal Amoltepec	1980	0
22	0_4	Santa Catarina Tayata	1980	125
23	5_9	Santa Catarina Tayata	1980	160
24	10_14	Santa Catarina Tayata	1980	112
25	15_19	Santa Catarina Tayata	1980	69
26	20_24	Santa Catarina Tayata	1980	68
27	25_29	Santa Catarina Tayata	1980	54
28	30_34	Santa Catarina Tayata	1980	41
29	35_39	Santa Catarina Tayata	1980	46
30	40_44	Santa Catarina Tayata	1980	39
31	45_49	Santa Catarina Tayata	1980	42
32	50_54	Santa Catarina Tayata	1980	27
33	55_59	Santa Catarina Tayata	1980	15

34	60_64	Santa Catarina Tayata	1980	18
35	65_69	Santa Catarina Tayata	1980	10
36	70_74	Santa Catarina Tayata	1980	8
37	75_79	Santa Catarina Tayata	1980	11
38	80_84	Santa Catarina Tayata	1980	7
39	85_89	Santa Catarina Tayata	1980	5
40	90_94	Santa Catarina Tayata	1980	1
41	95_99	Santa Catarina Tayata	1980	1
42	>100	Santa Catarina Tayata	1980	0
43	0_4	San Cristobal Amoltepec	1990	182
44	5_9	San Cristobal Amoltepec	1990	221
45	10_14	San Cristobal Amoltepec	1990	194
46	15_19	San Cristobal Amoltepec	1990	98
47	20_24	San Cristobal Amoltepec	1990	66
48	25_29	San Cristobal Amoltepec	1990	77
49	30_34	San Cristobal Amoltepec	1990	67
50	35_39	San Cristobal Amoltepec	1990	77
51	40_44	San Cristobal Amoltepec	1990	43
52	45_49	San Cristobal Amoltepec	1990	51
53	50_54	San Cristobal Amoltepec	1990	29
54	55_59	San Cristobal Amoltepec	1990	36
55	60_64	San Cristobal Amoltepec	1990	28
56	65_69	San Cristobal Amoltepec	1990	23
57	70_74	San Cristobal Amoltepec	1990	14
58	75_79	San Cristobal Amoltepec	1990	5
59	80_84	San Cristobal Amoltepec	1990	2
60	85_89	San Cristobal Amoltepec	1990	1
61	90_94	San Cristobal Amoltepec	1990	1
62	95_99	San Cristobal Amoltepec	1990	3
63	>100	San Cristobal Amoltepec	1990	1
64	0_4	Santa Catarina Tayata	1990	75
65	5_9	Santa Catarina Tayata	1990	124
66	10_14	Santa Catarina Tayata	1990	89
67	15_19	Santa Catarina Tayata	1990	76

68	20_24	Santa Catarina Tayata	1990	47
69	25_29	Santa Catarina Tayata	1990	41
70	30_34	Santa Catarina Tayata	1990	26
71	35_39	Santa Catarina Tayata	1990	30
72	40_44	Santa Catarina Tayata	1990	25
73	45_49	Santa Catarina Tayata	1990	26
74	50_54	Santa Catarina Tayata	1990	23
75	55_59	Santa Catarina Tayata	1990	28
76	60_64	Santa Catarina Tayata	1990	35
77	65_69	Santa Catarina Tayata	1990	32
78	70_74	Santa Catarina Tayata	1990	25
79	75_79	Santa Catarina Tayata	1990	19
80	80_84	Santa Catarina Tayata	1990	12
81	85_89	Santa Catarina Tayata	1990	8
82	90_94	Santa Catarina Tayata	1990	4
83	95_99	Santa Catarina Tayata	1990	1
84	>100	Santa Catarina Tayata	1990	1
85	0_4	San Cristobal Amoltepec	1995	152
86	5_9	San Cristobal Amoltepec	1995	175
87	10_14	San Cristobal Amoltepec	1995	188
88	15_19	San Cristobal Amoltepec	1995	113
89	20_24	San Cristobal Amoltepec	1995	72
90	25_29	San Cristobal Amoltepec	1995	58
91	30_34	San Cristobal Amoltepec	1995	54
92	35_39	San Cristobal Amoltepec	1995	47
93	40_44	San Cristobal Amoltepec	1995	47
94	45_49	San Cristobal Amoltepec	1995	41
95	50_54	San Cristobal Amoltepec	1995	26
96	55_59	San Cristobal Amoltepec	1995	37
97	60_64	San Cristobal Amoltepec	1995	28
98	65_69	San Cristobal Amoltepec	1995	17
99	70_74	San Cristobal Amoltepec	1995	8
100	75_79	San Cristobal Amoltepec	1995	7
101	80_84	San Cristobal Amoltepec	1995	1

102	85_89	San Cristobal Amoltepec	1995	1
103	90_94	San Cristobal Amoltepec	1995	1
104	95_99	San Cristobal Amoltepec	1995	0
105	>100	San Cristobal Amoltepec	1995	0
106	0_4	Santa Catarina Tayata	1995	79
107	5_9	Santa Catarina Tayata	1995	109
108	10_14	Santa Catarina Tayata	1995	116
109	15_19	Santa Catarina Tayata	1995	63
110	20_24	Santa Catarina Tayata	1995	61
111	25_29	Santa Catarina Tayata	1995	42
112	30_34	Santa Catarina Tayata	1995	44
113	35_39	Santa Catarina Tayata	1995	34
114	40_44	Santa Catarina Tayata	1995	22
115	45_49	Santa Catarina Tayata	1995	26
116	50_54	Santa Catarina Tayata	1995	24
117	55_59	Santa Catarina Tayata	1995	19
118	60_64	Santa Catarina Tayata	1995	27
119	65_69	Santa Catarina Tayata	1995	24
120	70_74	Santa Catarina Tayata	1995	24
121	75_79	Santa Catarina Tayata	1995	25
122	80_84	Santa Catarina Tayata	1995	18
123	85_89	Santa Catarina Tayata	1995	9
124	90_94	Santa Catarina Tayata	1995	2
125	95_99	Santa Catarina Tayata	1995	2
126	>100	Santa Catarina Tayata	1995	0
127	0_4	San Cristobal Amoltepec	2000	141
128	5_9	San Cristobal Amoltepec	2000	204
129	10_14	San Cristobal Amoltepec	2000	157
130	15_19	San Cristobal Amoltepec	2000	114
131	20_24	San Cristobal Amoltepec	2000	88
132	25_29	San Cristobal Amoltepec	2000	61
133	30_34	San Cristobal Amoltepec	2000	67
134	35_39	San Cristobal Amoltepec	2000	59
135	40_44	San Cristobal Amoltepec	2000	55

136	45_49	San Cristobal Amoltepec	2000	42
137	50_54	San Cristobal Amoltepec	2000	36
138	55_59	San Cristobal Amoltepec	2000	36
139	60_64	San Cristobal Amoltepec	2000	34
140	65_69	San Cristobal Amoltepec	2000	31
141	70_74	San Cristobal Amoltepec	2000	12
142	75_79	San Cristobal Amoltepec	2000	14
143	80_84	San Cristobal Amoltepec	2000	13
144	85_89	San Cristobal Amoltepec	2000	1
145	90_94	San Cristobal Amoltepec	2000	4
146	95_99	San Cristobal Amoltepec	2000	1
147	>100	San Cristobal Amoltepec	2000	0
148	0_4	Santa Catarina Tayata	2000	70
149	5_9	Santa Catarina Tayata	2000	75
150	10_14	Santa Catarina Tayata	2000	91
151	15_19	Santa Catarina Tayata	2000	85
152	20_24	Santa Catarina Tayata	2000	41
153	25_29	Santa Catarina Tayata	2000	43
154	30_34	Santa Catarina Tayata	2000	30
155	35_39	Santa Catarina Tayata	2000	36
156	40_44	Santa Catarina Tayata	2000	32
157	45_49	Santa Catarina Tayata	2000	27
158	50_54	Santa Catarina Tayata	2000	21
159	55_59	Santa Catarina Tayata	2000	20
160	60_64	Santa Catarina Tayata	2000	19
161	65_69	Santa Catarina Tayata	2000	21
162	70_74	Santa Catarina Tayata	2000	35
163	75_79	Santa Catarina Tayata	2000	26
164	80_84	Santa Catarina Tayata	2000	20
165	85_89	Santa Catarina Tayata	2000	10
166	90_94	Santa Catarina Tayata	2000	8
167	95_99	Santa Catarina Tayata	2000	4
168	>100	Santa Catarina Tayata	2000	1
169	0_4	San Cristobal Amoltepec	2005	131

170	5_9	San Cristobal Amoltepec	2005	122
171	10_14	San Cristobal Amoltepec	2005	179
172	15_19	San Cristobal Amoltepec	2005	119
173	20_24	San Cristobal Amoltepec	2005	99
174	25_29	San Cristobal Amoltepec	2005	91
175	30_34	San Cristobal Amoltepec	2005	57
176	35_39	San Cristobal Amoltepec	2005	65
177	40_44	San Cristobal Amoltepec	2005	51
178	45_49	San Cristobal Amoltepec	2005	49
179	50_54	San Cristobal Amoltepec	2005	52
180	55_59	San Cristobal Amoltepec	2005	32
181	60_64	San Cristobal Amoltepec	2005	30
182	65_69	San Cristobal Amoltepec	2005	36
183	70_74	San Cristobal Amoltepec	2005	17
184	75_79	San Cristobal Amoltepec	2005	12
185	80_84	San Cristobal Amoltepec	2005	16
186	85_89	San Cristobal Amoltepec	2005	11
187	90_94	San Cristobal Amoltepec	2005	0
188	95_99	San Cristobal Amoltepec	2005	0
189	>100	San Cristobal Amoltepec	2005	1
190	0_4	Santa Catarina Tayata	2005	76
191	5_9	Santa Catarina Tayata	2005	54
192	10_14	Santa Catarina Tayata	2005	57
193	15_19	Santa Catarina Tayata	2005	53
194	20_24	Santa Catarina Tayata	2005	44
195	25_29	Santa Catarina Tayata	2005	22
196	30_34	Santa Catarina Tayata	2005	41
197	35_39	Santa Catarina Tayata	2005	33
198	40_44	Santa Catarina Tayata	2005	27
199	45_49	Santa Catarina Tayata	2005	24
200	50_54	Santa Catarina Tayata	2005	28
201	55_59	Santa Catarina Tayata	2005	18
202	60_64	Santa Catarina Tayata	2005	24
203	65_69	Santa Catarina Tayata	2005	17

204	70_74	Santa Catarina Tayata	2005	18
205	75_79	Santa Catarina Tayata	2005	23
206	80_84	Santa Catarina Tayata	2005	13
207	85_89	Santa Catarina Tayata	2005	12
208	90_94	Santa Catarina Tayata	2005	1
209	95_99	Santa Catarina Tayata	2005	0
210	>100	Santa Catarina Tayata	2005	1
211	0_4	San Cristobal Amoltepec	2010	157
212	5_9	San Cristobal Amoltepec	2010	141
213	10_14	San Cristobal Amoltepec	2010	133
214	15_19	San Cristobal Amoltepec	2010	147
215	20_24	San Cristobal Amoltepec	2010	88
216	25_29	San Cristobal Amoltepec	2010	96
217	30_34	San Cristobal Amoltepec	2010	81
218	35_39	San Cristobal Amoltepec	2010	64
219	40_44	San Cristobal Amoltepec	2010	60
220	45_49	San Cristobal Amoltepec	2010	56
221	50_54	San Cristobal Amoltepec	2010	48
222	55_59	San Cristobal Amoltepec	2010	48
223	60_64	San Cristobal Amoltepec	2010	38
224	65_69	San Cristobal Amoltepec	2010	34
225	70_74	San Cristobal Amoltepec	2010	31
226	75_79	San Cristobal Amoltepec	2010	20
227	80_84	San Cristobal Amoltepec	2010	12
228	85_89	San Cristobal Amoltepec	2010	10
229	90_94	San Cristobal Amoltepec	2010	7
230	95_99	San Cristobal Amoltepec	2010	2
231	>100	San Cristobal Amoltepec	2010	1
232	0_4	Santa Catarina Tayata	2010	59
233	5_9	Santa Catarina Tayata	2010	85
234	10_14	Santa Catarina Tayata	2010	70
235	15_19	Santa Catarina Tayata	2010	57
236	20_24	Santa Catarina Tayata	2010	48
237	25_29	Santa Catarina Tayata	2010	43

238	30_34	Santa Catarina Tayata	2010	35
239	35_39	Santa Catarina Tayata	2010	45
240	40_44	Santa Catarina Tayata	2010	32
241	45_49	Santa Catarina Tayata	2010	28
242	50_54	Santa Catarina Tayata	2010	25
243	55_59	Santa Catarina Tayata	2010	29
244	60_64	Santa Catarina Tayata	2010	20
245	65_69	Santa Catarina Tayata	2010	17
246	70_74	Santa Catarina Tayata	2010	21
247	75_79	Santa Catarina Tayata	2010	26
248	80_84	Santa Catarina Tayata	2010	26
249	85_89	Santa Catarina Tayata	2010	11
250	90_94	Santa Catarina Tayata	2010	5
251	95_99	Santa Catarina Tayata	2010	3
252	>100	Santa Catarina Tayata	2010	2
