# Supplementary material for: The importance of the traditional milpa in food security and nutritional self-sufficiency in the highlands of Oaxaca, Mexico
Source: PLoS One. 2021 Feb 19;16(2):e0246281. doi: 10.1371/journal.pone.0246281 (PMC7894926; doi:10.1371/journal.pone.0246281)
Supplement: S3 Table — (PDF) [file pone.0246281.s003.pdf]

	year	community	Food source	nutrient/vitamin	Annual need	Percent of the population served
1	1991	Santa Catarina Tayata	blue tortilla	kcal	821250	8.08
2	2007	Santa Catarina Tayata	blue tortilla	kcal	821250	13.13
3	2018	Santa Catarina Tayata	blue tortilla	kcal	821250	18.61
4	1991	San Cristobal Amoltepec	blue tortilla	kcal	821250	15.14
5	2007	San Cristobal Amoltepec	blue tortilla	kcal	821250	9.27
6	2018	San Cristobal Amoltepec	blue tortilla	kcal	821250	12.51
7	1991	Santa Catarina Tayata	white tortilla	kcal	821250	59.08
8	2007	Santa Catarina Tayata	white tortilla	kcal	821250	96.07
9	2018	Santa Catarina Tayata	white tortilla	kcal	821250	136.17
10	1991	San Cristobal Amoltepec	white tortilla	kcal	821250	23.61
11	2007	San Cristobal Amoltepec	white tortilla	kcal	821250	14.46
12	2018	San Cristobal Amoltepec	white tortilla	kcal	821250	19.51
13	1991	Santa Catarina Tayata	yellow tortilla	kcal	821250	26.24
14	2007	Santa Catarina Tayata	yellow tortilla	kcal	821250	42.67
15	2018	Santa Catarina Tayata	yellow tortilla	kcal	821250	60.49
16	1991	San Cristobal Amoltepec	yellow tortilla	kcal	821250	24.74
17	2007	San Cristobal Amoltepec	yellow tortilla	kcal	821250	15.15
18	2018	San Cristobal Amoltepec	yellow tortilla	kcal	821250	20.45
19	1991	Santa Catarina Tayata	bean	kcal	821250	32.40
20	2007	Santa Catarina Tayata	bean	kcal	821250	34.86
21	2018	Santa Catarina Tayata	bean	kcal	821250	24.59
22	1991	San Cristobal Amoltepec	bean	kcal	821250	3.08
23	2007	San Cristobal Amoltepec	bean	kcal	821250	6.89
24	2018	San Cristobal Amoltepec	bean	kcal	821250	7.81
25	1991	Santa Catarina Tayata	squash flesh	kcal	821250	1.68
26	2007	Santa Catarina Tayata	squash flesh	kcal	821250	2.10
27	2018	Santa Catarina Tayata	squash flesh	kcal	821250	1.60
28	1991	San Cristobal Amoltepec	squash flesh	kcal	821250	0.27
29	2007	San Cristobal Amoltepec	squash flesh	kcal	821250	0.21
30	2018	San Cristobal Amoltepec	squash flesh	kcal	821250	0.27
31	1991	Santa Catarina Tayata	squash seeds	kcal	821250	1.84
32	2007	Santa Catarina Tayata	squash seeds	kcal	821250	2.29
33	2018	Santa Catarina Tayata	squash seeds	kcal	821250	1.74
34	1991	San Cristobal Amoltepec	squash seeds	kcal	821250	0.30
35	2007	San Cristobal Amoltepec	squash seeds	kcal	821250	0.22
36	2018	San Cristobal Amoltepec	squash seeds	kcal	821250	0.29
37	1991	Santa Catarina Tayata	fava	kcal	821250	0.50
38	2007	Santa Catarina Tayata	fava	kcal	821250	0.63
39	2018	Santa Catarina Tayata	fava	kcal	821250	0.48
40	1991	San Cristobal Amoltepec	fava	kcal	821250	0.33
41	2007	San Cristobal Amoltepec	fava	kcal	821250	0.25

**42**	2018	San Cristobal Amoltepec	fava	kcal	821250	0.33
**43**	1991	Santa Catarina Tayata	sheep	kcal	821250	6.26
**44**	2007	Santa Catarina Tayata	sheep	kcal	821250	10.25
**45**	2018	Santa Catarina Tayata	sheep	kcal	821250	14.12
**46**	1991	San Cristobal Amoltepec	sheep	kcal	821250	2.10
**47**	2007	San Cristobal Amoltepec	sheep	kcal	821250	2.96
**48**	2018	San Cristobal Amoltepec	sheep	kcal	821250	11.90
**49**	1991	Santa Catarina Tayata	blue tortilla	protein	18615	10.11
**50**	2007	Santa Catarina Tayata	blue tortilla	protein	18615	16.44
**51**	2018	Santa Catarina Tayata	blue tortilla	protein	18615	23.30
**52**	1991	San Cristobal Amoltepec	blue tortilla	protein	18615	18.95
**53**	2007	San Cristobal Amoltepec	blue tortilla	protein	18615	11.60
**54**	2018	San Cristobal Amoltepec	blue tortilla	protein	18615	15.66
**55**	1991	Santa Catarina Tayata	white tortilla	protein	18615	67.84
**56**	2007	Santa Catarina Tayata	white tortilla	protein	18615	110.31
**57**	2018	Santa Catarina Tayata	white tortilla	protein	18615	156.36
**58**	1991	San Cristobal Amoltepec	white tortilla	protein	18615	27.11
**59**	2007	San Cristobal Amoltepec	white tortilla	protein	18615	16.60
**60**	2018	San Cristobal Amoltepec	white tortilla	protein	18615	22.41
**61**	1991	Santa Catarina Tayata	yellow tortilla	protein	18615	30.13
**62**	2007	Santa Catarina Tayata	yellow tortilla	protein	18615	49.00
**63**	2018	Santa Catarina Tayata	yellow tortilla	protein	18615	69.45
**64**	1991	San Cristobal Amoltepec	yellow tortilla	protein	18615	28.41
**65**	2007	San Cristobal Amoltepec	yellow tortilla	protein	18615	17.40
**66**	2018	San Cristobal Amoltepec	yellow tortilla	protein	18615	23.48
**67**	1991	Santa Catarina Tayata	bean	protein	18615	86.52
**68**	2007	Santa Catarina Tayata	bean	protein	18615	93.08
**69**	2018	Santa Catarina Tayata	bean	protein	18615	65.66
**70**	1991	San Cristobal Amoltepec	bean	protein	18615	8.22
**71**	2007	San Cristobal Amoltepec	bean	protein	18615	18.40
**72**	2018	San Cristobal Amoltepec	bean	protein	18615	20.86
**73**	1991	Santa Catarina Tayata	squash flesh	protein	18615	1.48
**74**	2007	Santa Catarina Tayata	squash flesh	protein	18615	1.85
**75**	2018	Santa Catarina Tayata	squash flesh	protein	18615	1.41
**76**	1991	San Cristobal Amoltepec	squash flesh	protein	18615	0.24
**77**	2007	San Cristobal Amoltepec	squash flesh	protein	18615	0.18
**78**	2018	San Cristobal Amoltepec	squash flesh	protein	18615	0.24
**79**	1991	Santa Catarina Tayata	squash seeds	protein	18615	4.24
**80**	2007	Santa Catarina Tayata	squash seeds	protein	18615	5.29
**81**	2018	Santa Catarina Tayata	squash seeds	protein	18615	4.02
**82**	1991	San Cristobal Amoltepec	squash seeds	protein	18615	0.69
**83**	2007	San Cristobal Amoltepec	squash seeds	protein	18615	0.52
**84**	2018	San Cristobal Amoltepec	squash seeds	protein	18615	0.68

85	1991	Santa Catarina Tayata	fava	protein	18615	1.70
86	2007	Santa Catarina Tayata	fava	protein	18615	2.11
87	2018	Santa Catarina Tayata	fava	protein	18615	1.61
88	1991	San Cristobal Amoltepec	fava	protein	18615	1.13
89	2007	San Cristobal Amoltepec	fava	protein	18615	0.84
90	2018	San Cristobal Amoltepec	fava	protein	18615	1.10
91	1991	Santa Catarina Tayata	sheep	protein	18615	17.46
92	2007	Santa Catarina Tayata	sheep	protein	18615	28.60
93	2018	Santa Catarina Tayata	sheep	protein	18615	39.39
94	1991	San Cristobal Amoltepec	sheep	protein	18615	5.86
95	2007	San Cristobal Amoltepec	sheep	protein	18615	8.26
96	2018	San Cristobal Amoltepec	sheep	protein	18615	33.19
97	1991	Santa Catarina Tayata	blue tortilla	calcium	365000	11.45
98	2007	Santa Catarina Tayata	blue tortilla	calcium	365000	18.63
99	2018	Santa Catarina Tayata	blue tortilla	calcium	365000	26.40
100	1991	San Cristobal Amoltepec	blue tortilla	calcium	365000	21.48
101	2007	San Cristobal Amoltepec	blue tortilla	calcium	365000	13.15
102	2018	San Cristobal Amoltepec	blue tortilla	calcium	365000	17.75
103	1991	Santa Catarina Tayata	white tortilla	calcium	365000	49.16
104	2007	Santa Catarina Tayata	white tortilla	calcium	365000	79.95
105	2018	Santa Catarina Tayata	white tortilla	calcium	365000	113.32
106	1991	San Cristobal Amoltepec	white tortilla	calcium	365000	19.65
107	2007	San Cristobal Amoltepec	white tortilla	calcium	365000	12.03
108	2018	San Cristobal Amoltepec	white tortilla	calcium	365000	16.24
109	1991	Santa Catarina Tayata	yellow tortilla	calcium	365000	21.84
110	2007	Santa Catarina Tayata	yellow tortilla	calcium	365000	35.51
111	2018	Santa Catarina Tayata	yellow tortilla	calcium	365000	50.34
112	1991	San Cristobal Amoltepec	yellow tortilla	calcium	365000	20.59
113	2007	San Cristobal Amoltepec	yellow tortilla	calcium	365000	12.61
114	2018	San Cristobal Amoltepec	yellow tortilla	calcium	365000	17.02
115	1991	Santa Catarina Tayata	bean	calcium	365000	28.15
116	2007	Santa Catarina Tayata	bean	calcium	365000	30.29
117	2018	Santa Catarina Tayata	bean	calcium	365000	21.37
118	1991	San Cristobal Amoltepec	bean	calcium	365000	2.68
119	2007	San Cristobal Amoltepec	bean	calcium	365000	5.99
120	2018	San Cristobal Amoltepec	bean	calcium	365000	6.79
121	1991	Santa Catarina Tayata	squash flesh	calcium	365000	2.40
122	2007	Santa Catarina Tayata	squash flesh	calcium	365000	2.99
123	2018	Santa Catarina Tayata	squash flesh	calcium	365000	2.27
124	1991	San Cristobal Amoltepec	squash flesh	calcium	365000	0.39
125	2007	San Cristobal Amoltepec	squash flesh	calcium	365000	0.29
126	2018	San Cristobal Amoltepec	squash flesh	calcium	365000	0.38
127	1991	Santa Catarina Tayata	squash seeds	calcium	365000	0.38

128	2007	Santa Catarina Tayata	squash seeds	calcium	365000	0.47
129	2018	Santa Catarina Tayata	squash seeds	calcium	365000	0.36
130	1991	San Cristobal Amoltepec	squash seeds	calcium	365000	0.06
131	2007	San Cristobal Amoltepec	squash seeds	calcium	365000	0.05
132	2018	San Cristobal Amoltepec	squash seeds	calcium	365000	0.06
133	1991	Santa Catarina Tayata	fava	calcium	365000	0.34
134	2007	Santa Catarina Tayata	fava	calcium	365000	0.43
135	2018	Santa Catarina Tayata	fava	calcium	365000	0.32
136	1991	San Cristobal Amoltepec	fava	calcium	365000	0.23
137	2007	San Cristobal Amoltepec	fava	calcium	365000	0.17
138	2018	San Cristobal Amoltepec	fava	calcium	365000	0.22
139	1991	Santa Catarina Tayata	sheep	calcium	365000	0.63
140	2007	Santa Catarina Tayata	sheep	calcium	365000	1.04
141	2018	Santa Catarina Tayata	sheep	calcium	365000	1.43
142	1991	San Cristobal Amoltepec	sheep	calcium	365000	0.21
143	2007	San Cristobal Amoltepec	sheep	calcium	365000	0.30
144	2018	San Cristobal Amoltepec	sheep	calcium	365000	1.20
145	1991	Santa Catarina Tayata	blue tortilla	phosphorus	255500	31.88
146	2007	Santa Catarina Tayata	blue tortilla	phosphorus	255500	51.84
147	2018	Santa Catarina Tayata	blue tortilla	phosphorus	255500	73.48
148	1991	San Cristobal Amoltepec	blue tortilla	phosphorus	255500	59.78
149	2007	San Cristobal Amoltepec	blue tortilla	phosphorus	255500	36.60
150	2018	San Cristobal Amoltepec	blue tortilla	phosphorus	255500	49.41
151	1991	Santa Catarina Tayata	white tortilla	phosphorus	255500	70.23
152	2007	Santa Catarina Tayata	white tortilla	phosphorus	255500	114.21
153	2018	Santa Catarina Tayata	white tortilla	phosphorus	255500	161.89
154	1991	San Cristobal Amoltepec	white tortilla	phosphorus	255500	28.07
155	2007	San Cristobal Amoltepec	white tortilla	phosphorus	255500	17.18
156	2018	San Cristobal Amoltepec	white tortilla	phosphorus	255500	23.20
157	1991	Santa Catarina Tayata	yellow tortilla	phosphorus	255500	31.20
158	2007	Santa Catarina Tayata	yellow tortilla	phosphorus	255500	50.73
159	2018	Santa Catarina Tayata	yellow tortilla	phosphorus	255500	71.91
160	1991	San Cristobal Amoltepec	yellow tortilla	phosphorus	255500	29.42
161	2007	San Cristobal Amoltepec	yellow tortilla	phosphorus	255500	18.01
162	2018	San Cristobal Amoltepec	yellow tortilla	phosphorus	255500	24.31
163	1991	Santa Catarina Tayata	bean	phosphorus	255500	124.56
164	2007	Santa Catarina Tayata	bean	phosphorus	255500	134.01
165	2018	Santa Catarina Tayata	bean	phosphorus	255500	94.54
166	1991	San Cristobal Amoltepec	bean	phosphorus	255500	11.84
167	2007	San Cristobal Amoltepec	bean	phosphorus	255500	26.49
168	2018	San Cristobal Amoltepec	bean	phosphorus	255500	30.03
169	1991	Santa Catarina Tayata	squash flesh	phosphorus	255500	3.97
170	2007	Santa Catarina Tayata	squash flesh	phosphorus	255500	4.94

171	2018	Santa Catarina Tayata	squash flesh	phosphorus	255500	3.76
172	1991	San Cristobal Amoltepec	squash flesh	phosphorus	255500	0.65
173	2007	San Cristobal Amoltepec	squash flesh	phosphorus	255500	0.48
174	2018	San Cristobal Amoltepec	squash flesh	phosphorus	255500	0.63
175	1991	Santa Catarina Tayata	squash seeds	phosphorus	255500	12.10
176	2007	Santa Catarina Tayata	squash seeds	phosphorus	255500	15.07
177	2018	Santa Catarina Tayata	squash seeds	phosphorus	255500	11.47
178	1991	San Cristobal Amoltepec	squash seeds	phosphorus	255500	1.98
179	2007	San Cristobal Amoltepec	squash seeds	phosphorus	255500	1.48
180	2018	San Cristobal Amoltepec	squash seeds	phosphorus	255500	1.94
181	1991	Santa Catarina Tayata	fava	phosphorus	255500	2.00
182	2007	Santa Catarina Tayata	fava	phosphorus	255500	2.49
183	2018	Santa Catarina Tayata	fava	phosphorus	255500	1.90
184	1991	San Cristobal Amoltepec	fava	phosphorus	255500	1.33
185	2007	San Cristobal Amoltepec	fava	phosphorus	255500	0.99
186	2018	San Cristobal Amoltepec	fava	phosphorus	255500	1.30
187	1991	Santa Catarina Tayata	sheep	phosphorus	255500	12.06
188	2007	Santa Catarina Tayata	sheep	phosphorus	255500	19.75
189	2018	Santa Catarina Tayata	sheep	phosphorus	255500	27.20
190	1991	San Cristobal Amoltepec	sheep	phosphorus	255500	4.05
191	2007	San Cristobal Amoltepec	sheep	phosphorus	255500	5.70
192	2018	San Cristobal Amoltepec	sheep	phosphorus	255500	22.92
193	1991	Santa Catarina Tayata	blue tortilla	iron	6570	10.57
194	2007	Santa Catarina Tayata	blue tortilla	iron	6570	17.19
195	2018	Santa Catarina Tayata	blue tortilla	iron	6570	24.36
196	1991	San Cristobal Amoltepec	blue tortilla	iron	6570	19.82
197	2007	San Cristobal Amoltepec	blue tortilla	iron	6570	12.13
198	2018	San Cristobal Amoltepec	blue tortilla	iron	6570	16.38
199	1991	Santa Catarina Tayata	white tortilla	iron	6570	40.46
200	2007	Santa Catarina Tayata	white tortilla	iron	6570	65.80
201	2018	Santa Catarina Tayata	white tortilla	iron	6570	93.27
202	1991	San Cristobal Amoltepec	white tortilla	iron	6570	16.17
203	2007	San Cristobal Amoltepec	white tortilla	iron	6570	9.90
204	2018	San Cristobal Amoltepec	white tortilla	iron	6570	13.36
205	1991	Santa Catarina Tayata	yellow tortilla	iron	6570	17.97
206	2007	Santa Catarina Tayata	yellow tortilla	iron	6570	29.23
207	2018	Santa Catarina Tayata	yellow tortilla	iron	6570	41.43
208	1991	San Cristobal Amoltepec	yellow tortilla	iron	6570	16.95
209	2007	San Cristobal Amoltepec	yellow tortilla	iron	6570	10.38
210	2018	San Cristobal Amoltepec	yellow tortilla	iron	6570	14.01
211	1991	Santa Catarina Tayata	bean	iron	6570	82.88
212	2007	Santa Catarina Tayata	bean	iron	6570	89.16
213	2018	Santa Catarina Tayata	bean	iron	6570	62.90

214	1991	San Cristobal Amoltepec	bean	iron	6570	7.87
215	2007	San Cristobal Amoltepec	bean	iron	6570	17.63
216	2018	San Cristobal Amoltepec	bean	iron	6570	19.98
217	1991	Santa Catarina Tayata	squash flesh	iron	6570	3.25
218	2007	Santa Catarina Tayata	squash flesh	iron	6570	4.04
219	2018	Santa Catarina Tayata	squash flesh	iron	6570	3.08
220	1991	San Cristobal Amoltepec	squash flesh	iron	6570	0.53
221	2007	San Cristobal Amoltepec	squash flesh	iron	6570	0.40
222	2018	San Cristobal Amoltepec	squash flesh	iron	6570	0.52
223	1991	Santa Catarina Tayata	squash seeds	iron	6570	1.24
224	2007	Santa Catarina Tayata	squash seeds	iron	6570	1.54
225	2018	Santa Catarina Tayata	squash seeds	iron	6570	1.17
226	1991	San Cristobal Amoltepec	squash seeds	iron	6570	0.82
227	2007	San Cristobal Amoltepec	squash seeds	iron	6570	0.61
228	2018	San Cristobal Amoltepec	squash seeds	iron	6570	0.81
229	1991	Santa Catarina Tayata	fava	iron	6570	1.24
230	2007	Santa Catarina Tayata	fava	iron	6570	1.54
231	2018	Santa Catarina Tayata	fava	iron	6570	1.17
232	1991	San Cristobal Amoltepec	fava	iron	6570	0.82
233	2007	San Cristobal Amoltepec	fava	iron	6570	0.61
234	2018	San Cristobal Amoltepec	fava	iron	6570	0.81
235	1991	Santa Catarina Tayata	sheep	iron	6570	4.60
236	2007	Santa Catarina Tayata	sheep	iron	6570	7.54
237	2018	Santa Catarina Tayata	sheep	iron	6570	10.38
238	1991	San Cristobal Amoltepec	sheep	iron	6570	1.54
239	2007	San Cristobal Amoltepec	sheep	iron	6570	2.18
240	2018	San Cristobal Amoltepec	sheep	iron	6570	8.75
241	1991	Santa Catarina Tayata	blue tortilla	magnesium	144905	5.31
242	2007	Santa Catarina Tayata	blue tortilla	magnesium	144905	8.63
243	2018	Santa Catarina Tayata	blue tortilla	magnesium	144905	12.23
244	1991	San Cristobal Amoltepec	blue tortilla	magnesium	144905	9.95
245	2007	San Cristobal Amoltepec	blue tortilla	magnesium	144905	6.09
246	2018	San Cristobal Amoltepec	blue tortilla	magnesium	144905	8.22
247	1991	Santa Catarina Tayata	white tortilla	magnesium	144905	110.08
248	2007	Santa Catarina Tayata	white tortilla	magnesium	144905	179.00
249	2018	Santa Catarina Tayata	white tortilla	magnesium	144905	253.73
250	1991	San Cristobal Amoltepec	white tortilla	magnesium	144905	43.99
251	2007	San Cristobal Amoltepec	white tortilla	magnesium	144905	26.93
252	2018	San Cristobal Amoltepec	white tortilla	magnesium	144905	36.36
253	1991	Santa Catarina Tayata	yellow tortilla	magnesium	144905	48.89
254	2007	Santa Catarina Tayata	yellow tortilla	magnesium	144905	79.51
255	2018	Santa Catarina Tayata	yellow tortilla	magnesium	144905	112.70
256	1991	San Cristobal Amoltepec	yellow tortilla	magnesium	144905	46.11

257	2007	San Cristobal Amoltepec	yellow tortilla	magnesium	144905	28.23
258	2018	San Cristobal Amoltepec	yellow tortilla	magnesium	144905	38.11
259	1991	Santa Catarina Tayata	bean	magnesium	144905	31.75
260	2007	Santa Catarina Tayata	bean	magnesium	144905	34.16
261	2018	Santa Catarina Tayata	bean	magnesium	144905	24.10
262	1991	San Cristobal Amoltepec	bean	magnesium	144905	3.02
263	2007	San Cristobal Amoltepec	bean	magnesium	144905	6.75
264	2018	San Cristobal Amoltepec	bean	magnesium	144905	7.65
265	1991	Santa Catarina Tayata	squash flesh	magnesium	144905	7.95
266	2007	Santa Catarina Tayata	squash flesh	magnesium	144905	9.90
267	2018	Santa Catarina Tayata	squash flesh	magnesium	144905	7.54
268	1991	San Cristobal Amoltepec	squash flesh	magnesium	144905	1.30
269	2007	San Cristobal Amoltepec	squash flesh	magnesium	144905	0.97
270	2018	San Cristobal Amoltepec	squash flesh	magnesium	144905	1.27
271	1991	Santa Catarina Tayata	squash seeds	magnesium	144905	9.99
272	2007	Santa Catarina Tayata	squash seeds	magnesium	144905	12.45
273	2018	Santa Catarina Tayata	squash seeds	magnesium	144905	9.47
274	1991	San Cristobal Amoltepec	squash seeds	magnesium	144905	1.63
275	2007	San Cristobal Amoltepec	squash seeds	magnesium	144905	1.22
276	2018	San Cristobal Amoltepec	squash seeds	magnesium	144905	1.60
277	1991	Santa Catarina Tayata	fava	magnesium	144905	1.61
278	2007	Santa Catarina Tayata	fava	magnesium	144905	2.00
279	2018	Santa Catarina Tayata	fava	magnesium	144905	1.53
280	1991	San Cristobal Amoltepec	fava	magnesium	144905	1.07
281	2007	San Cristobal Amoltepec	fava	magnesium	144905	0.80
282	2018	San Cristobal Amoltepec	fava	magnesium	144905	1.05
283	1991	Santa Catarina Tayata	sheep	magnesium	144905	2.92
284	2007	Santa Catarina Tayata	sheep	magnesium	144905	4.79
285	2018	Santa Catarina Tayata	sheep	magnesium	144905	6.60
286	1991	San Cristobal Amoltepec	sheep	magnesium	144905	0.98
287	2007	San Cristobal Amoltepec	sheep	magnesium	144905	1.38
288	2018	San Cristobal Amoltepec	sheep	magnesium	144905	5.56
289	1991	Santa Catarina Tayata	blue tortilla	zinc	3650	4.48
290	2007	Santa Catarina Tayata	blue tortilla	zinc	3650	7.28
291	2018	Santa Catarina Tayata	blue tortilla	zinc	3650	10.32
292	1991	San Cristobal Amoltepec	blue tortilla	zinc	3650	8.39
293	2007	San Cristobal Amoltepec	blue tortilla	zinc	3650	5.14
294	2018	San Cristobal Amoltepec	blue tortilla	zinc	3650	6.94
295	1991	Santa Catarina Tayata	white tortilla	zinc	3650	12.14
296	2007	Santa Catarina Tayata	white tortilla	zinc	3650	19.74
297	2018	Santa Catarina Tayata	white tortilla	zinc	3650	27.98
298	1991	San Cristobal Amoltepec	white tortilla	zinc	3650	4.85
299	2007	San Cristobal Amoltepec	white tortilla	zinc	3650	2.97

300	2018	San Cristobal Amoltepec	white tortilla	zinc	3650	4.01
301	1991	Santa Catarina Tayata	yellow tortilla	zinc	3650	5.39
302	2007	Santa Catarina Tayata	yellow tortilla	zinc	3650	8.77
303	2018	Santa Catarina Tayata	yellow tortilla	zinc	3650	12.43
304	1991	San Cristobal Amoltepec	yellow tortilla	zinc	3650	5.08
305	2007	San Cristobal Amoltepec	yellow tortilla	zinc	3650	3.11
306	2018	San Cristobal Amoltepec	yellow tortilla	zinc	3650	4.20
307	1991	Santa Catarina Tayata	bean	zinc	3650	53.58
308	2007	Santa Catarina Tayata	bean	zinc	3650	57.64
309	2018	Santa Catarina Tayata	bean	zinc	3650	40.66
310	1991	San Cristobal Amoltepec	bean	zinc	3650	5.09
311	2007	San Cristobal Amoltepec	bean	zinc	3650	11.39
312	2018	San Cristobal Amoltepec	bean	zinc	3650	12.92
313	1991	Santa Catarina Tayata	squash flesh	zinc	3650	4.04
314	2007	Santa Catarina Tayata	squash flesh	zinc	3650	5.03
315	2018	Santa Catarina Tayata	squash flesh	zinc	3650	3.83
316	1991	San Cristobal Amoltepec	squash flesh	zinc	3650	0.66
317	2007	San Cristobal Amoltepec	squash flesh	zinc	3650	0.49
318	2018	San Cristobal Amoltepec	squash flesh	zinc	3650	0.65
319	1991	Santa Catarina Tayata	squash seeds	zinc	3650	5.51
320	2007	Santa Catarina Tayata	squash seeds	zinc	3650	6.87
321	2018	Santa Catarina Tayata	squash seeds	zinc	3650	5.22
322	1991	San Cristobal Amoltepec	squash seeds	zinc	3650	0.90
323	2007	San Cristobal Amoltepec	squash seeds	zinc	3650	0.67
324	2018	San Cristobal Amoltepec	squash seeds	zinc	3650	0.88
325	1991	Santa Catarina Tayata	fava	zinc	3650	1.04
326	2007	Santa Catarina Tayata	fava	zinc	3650	1.30
327	2018	Santa Catarina Tayata	fava	zinc	3650	0.99
328	1991	San Cristobal Amoltepec	fava	zinc	3650	0.69
329	2007	San Cristobal Amoltepec	fava	zinc	3650	0.52
330	2018	San Cristobal Amoltepec	fava	zinc	3650	0.68
331	1991	Santa Catarina Tayata	sheep	zinc	3650	17.56
332	2007	Santa Catarina Tayata	sheep	zinc	3650	28.77
333	2018	Santa Catarina Tayata	sheep	zinc	3650	39.63
334	1991	San Cristobal Amoltepec	sheep	zinc	3650	5.90
335	2007	San Cristobal Amoltepec	sheep	zinc	3650	8.31
336	2018	San Cristobal Amoltepec	sheep	zinc	3650	33.39
337	1991	Santa Catarina Tayata	blue tortilla	vitamin c	29200	0.00
338	2007	Santa Catarina Tayata	blue tortilla	vitamin c	29200	0.00
339	2018	Santa Catarina Tayata	blue tortilla	vitamin c	29200	0.00
340	1991	San Cristobal Amoltepec	blue tortilla	vitamin c	29200	0.00
341	2007	San Cristobal Amoltepec	blue tortilla	vitamin c	29200	0.00
342	2018	San Cristobal Amoltepec	blue tortilla	vitamin c	29200	0.00

343	1991	Santa Catarina Tayata	white tortilla	vitamin c	29200	0.00
344	2007	Santa Catarina Tayata	white tortilla	vitamin c	29200	0.00
345	2018	Santa Catarina Tayata	white tortilla	vitamin c	29200	0.00
346	1991	San Cristobal Amoltepec	white tortilla	vitamin c	29200	0.00
347	2007	San Cristobal Amoltepec	white tortilla	vitamin c	29200	0.00
348	2018	San Cristobal Amoltepec	white tortilla	vitamin c	29200	0.00
349	1991	Santa Catarina Tayata	yellow tortilla	vitamin c	29200	0.00
350	2007	Santa Catarina Tayata	yellow tortilla	vitamin c	29200	0.00
351	2018	Santa Catarina Tayata	yellow tortilla	vitamin c	29200	0.00
352	1991	San Cristobal Amoltepec	yellow tortilla	vitamin c	29200	0.00
353	2007	San Cristobal Amoltepec	yellow tortilla	vitamin c	29200	0.00
354	2018	San Cristobal Amoltepec	yellow tortilla	vitamin c	29200	0.00
355	1991	Santa Catarina Tayata	bean	vitamin c	29200	2.63
356	2007	Santa Catarina Tayata	bean	vitamin c	29200	2.83
357	2018	Santa Catarina Tayata	bean	vitamin c	29200	1.99
358	1991	San Cristobal Amoltepec	bean	vitamin c	29200	0.25
359	2007	San Cristobal Amoltepec	bean	vitamin c	29200	0.56
360	2018	San Cristobal Amoltepec	bean	vitamin c	29200	0.63
361	1991	Santa Catarina Tayata	squash flesh	vitamin c	29200	23.66
362	2007	Santa Catarina Tayata	squash flesh	vitamin c	29200	29.49
363	2018	Santa Catarina Tayata	squash flesh	vitamin c	29200	22.44
364	1991	San Cristobal Amoltepec	squash flesh	vitamin c	29200	3.87
365	2007	San Cristobal Amoltepec	squash flesh	vitamin c	29200	2.89
366	2018	San Cristobal Amoltepec	squash flesh	vitamin c	29200	3.79
367	1991	Santa Catarina Tayata	squash seeds	vitamin c	29200	0.16
368	2007	Santa Catarina Tayata	squash seeds	vitamin c	29200	0.20
369	2018	Santa Catarina Tayata	squash seeds	vitamin c	29200	0.15
370	1991	San Cristobal Amoltepec	squash seeds	vitamin c	29200	0.03
371	2007	San Cristobal Amoltepec	squash seeds	vitamin c	29200	0.02
372	2018	San Cristobal Amoltepec	squash seeds	vitamin c	29200	0.03
373	1991	Santa Catarina Tayata	fava	vitamin c	29200	0.04
374	2007	Santa Catarina Tayata	fava	vitamin c	29200	0.05
375	2018	Santa Catarina Tayata	fava	vitamin c	29200	0.04
376	1991	San Cristobal Amoltepec	fava	vitamin c	29200	0.03
377	2007	San Cristobal Amoltepec	fava	vitamin c	29200	0.02
378	2018	San Cristobal Amoltepec	fava	vitamin c	29200	0.03
379	1991	Santa Catarina Tayata	sheep	vitamin c	29200	0.00
380	2007	Santa Catarina Tayata	sheep	vitamin c	29200	0.00
381	2018	Santa Catarina Tayata	sheep	vitamin c	29200	0.00
382	1991	San Cristobal Amoltepec	sheep	vitamin c	29200	0.00
383	2007	San Cristobal Amoltepec	sheep	vitamin c	29200	0.00
384	2018	San Cristobal Amoltepec	sheep	vitamin c	29200	0.00
385	1991	Santa Catarina Tayata	blue tortilla	vitamin a	292000	0.16

386	2007	Santa Catarina Tayata	blue tortilla	vitamin a	292000	0.27
387	2018	Santa Catarina Tayata	blue tortilla	vitamin a	292000	0.38
388	1991	San Cristobal Amoltepec	blue tortilla	vitamin a	292000	0.31
389	2007	San Cristobal Amoltepec	blue tortilla	vitamin a	292000	0.19
390	2018	San Cristobal Amoltepec	blue tortilla	vitamin a	292000	0.26
391	1991	Santa Catarina Tayata	white tortilla	vitamin a	292000	0.00
392	2007	Santa Catarina Tayata	white tortilla	vitamin a	292000	0.00
393	2018	Santa Catarina Tayata	white tortilla	vitamin a	292000	0.00
394	1991	San Cristobal Amoltepec	white tortilla	vitamin a	292000	0.00
395	2007	San Cristobal Amoltepec	white tortilla	vitamin a	292000	0.00
396	2018	San Cristobal Amoltepec	white tortilla	vitamin a	292000	0.00
397	1991	Santa Catarina Tayata	yellow tortilla	vitamin a	292000	0.00
398	2007	Santa Catarina Tayata	yellow tortilla	vitamin a	292000	0.00
399	2018	Santa Catarina Tayata	yellow tortilla	vitamin a	292000	0.00
400	1991	San Cristobal Amoltepec	yellow tortilla	vitamin a	292000	0.00
401	2007	San Cristobal Amoltepec	yellow tortilla	vitamin a	292000	0.00
402	2018	San Cristobal Amoltepec	yellow tortilla	vitamin a	292000	0.00
403	1991	Santa Catarina Tayata	bean	vitamin a	292000	0.00
404	2007	Santa Catarina Tayata	bean	vitamin a	292000	0.00
405	2018	Santa Catarina Tayata	bean	vitamin a	292000	0.00
406	1991	San Cristobal Amoltepec	bean	vitamin a	292000	0.00
407	2007	San Cristobal Amoltepec	bean	vitamin a	292000	0.00
408	2018	San Cristobal Amoltepec	bean	vitamin a	292000	0.00
409	1991	Santa Catarina Tayata	squash flesh	vitamin a	292000	22.56
410	2007	Santa Catarina Tayata	squash flesh	vitamin a	292000	28.12
411	2018	Santa Catarina Tayata	squash flesh	vitamin a	292000	21.39
412	1991	San Cristobal Amoltepec	squash flesh	vitamin a	292000	3.69
413	2007	San Cristobal Amoltepec	squash flesh	vitamin a	292000	2.75
414	2018	San Cristobal Amoltepec	squash flesh	vitamin a	292000	3.61
415	1991	Santa Catarina Tayata	squash seeds	vitamin a	292000	0.00
416	2007	Santa Catarina Tayata	squash seeds	vitamin a	292000	0.00
417	2018	Santa Catarina Tayata	squash seeds	vitamin a	292000	0.00
418	1991	San Cristobal Amoltepec	squash seeds	vitamin a	292000	0.00
419	2007	San Cristobal Amoltepec	squash seeds	vitamin a	292000	0.00
420	2018	San Cristobal Amoltepec	squash seeds	vitamin a	292000	0.00
421	1991	Santa Catarina Tayata	fava	vitamin a	292000	0.01
422	2007	Santa Catarina Tayata	fava	vitamin a	292000	0.02
423	2018	Santa Catarina Tayata	fava	vitamin a	292000	0.01
424	1991	San Cristobal Amoltepec	fava	vitamin a	292000	0.01
425	2007	San Cristobal Amoltepec	fava	vitamin a	292000	0.01
426	2018	San Cristobal Amoltepec	fava	vitamin a	292000	0.01
427	1991	Santa Catarina Tayata	sheep	vitamin a	292000	0.00
428	2007	Santa Catarina Tayata	sheep	vitamin a	292000	0.00

429	2018	Santa Catarina Tayata	sheep	vitamin a	292000	0.00
430	1991	San Cristobal Amoltepec	sheep	vitamin a	292000	0.00
431	2007	San Cristobal Amoltepec	sheep	vitamin a	292000	0.00
432	2018	San Cristobal Amoltepec	sheep	vitamin a	292000	0.00
433	1991	Santa Catarina Tayata	blue tortilla	vitamin b6	474.5	5.06
434	2007	Santa Catarina Tayata	blue tortilla	vitamin b6	474.5	8.23
435	2018	Santa Catarina Tayata	blue tortilla	vitamin b6	474.5	11.67
436	1991	San Cristobal Amoltepec	blue tortilla	vitamin b6	474.5	9.49
437	2007	San Cristobal Amoltepec	blue tortilla	vitamin b6	474.5	5.81
438	2018	San Cristobal Amoltepec	blue tortilla	vitamin b6	474.5	7.85
439	1991	Santa Catarina Tayata	white tortilla	vitamin b6	474.5	93.38
440	2007	Santa Catarina Tayata	white tortilla	vitamin b6	474.5	151.85
441	2018	Santa Catarina Tayata	white tortilla	vitamin b6	474.5	215.24
442	1991	San Cristobal Amoltepec	white tortilla	vitamin b6	474.5	37.31
443	2007	San Cristobal Amoltepec	white tortilla	vitamin b6	474.5	22.85
444	2018	San Cristobal Amoltepec	white tortilla	vitamin b6	474.5	30.84
445	1991	Santa Catarina Tayata	yellow tortilla	vitamin b6	474.5	41.48
446	2007	Santa Catarina Tayata	yellow tortilla	vitamin b6	474.5	67.45
447	2018	Santa Catarina Tayata	yellow tortilla	vitamin b6	474.5	95.60
448	1991	San Cristobal Amoltepec	yellow tortilla	vitamin b6	474.5	39.11
449	2007	San Cristobal Amoltepec	yellow tortilla	vitamin b6	474.5	23.95
450	2018	San Cristobal Amoltepec	yellow tortilla	vitamin b6	474.5	32.33
451	1991	Santa Catarina Tayata	bean	vitamin b6	474.5	85.66
452	2007	Santa Catarina Tayata	bean	vitamin b6	474.5	92.16
453	2018	Santa Catarina Tayata	bean	vitamin b6	474.5	65.01
454	1991	San Cristobal Amoltepec	bean	vitamin b6	474.5	8.14
455	2007	San Cristobal Amoltepec	bean	vitamin b6	474.5	18.22
456	2018	San Cristobal Amoltepec	bean	vitamin b6	474.5	20.65
457	1991	Santa Catarina Tayata	squash flesh	vitamin b6	474.5	5.82
458	2007	Santa Catarina Tayata	squash flesh	vitamin b6	474.5	7.26
459	2018	Santa Catarina Tayata	squash flesh	vitamin b6	474.5	5.52
460	1991	San Cristobal Amoltepec	squash flesh	vitamin b6	474.5	0.95
461	2007	San Cristobal Amoltepec	squash flesh	vitamin b6	474.5	0.71
462	2018	San Cristobal Amoltepec	squash flesh	vitamin b6	474.5	0.93
463	1991	Santa Catarina Tayata	squash seeds	vitamin b6	474.5	0.55
464	2007	Santa Catarina Tayata	squash seeds	vitamin b6	474.5	0.69
465	2018	Santa Catarina Tayata	squash seeds	vitamin b6	474.5	0.53
466	1991	San Cristobal Amoltepec	squash seeds	vitamin b6	474.5	0.09
467	2007	San Cristobal Amoltepec	squash seeds	vitamin b6	474.5	0.07
468	2018	San Cristobal Amoltepec	squash seeds	vitamin b6	474.5	0.09
469	1991	Santa Catarina Tayata	fava	vitamin b6	474.5	0.95
470	2007	Santa Catarina Tayata	fava	vitamin b6	474.5	1.18
471	2018	Santa Catarina Tayata	fava	vitamin b6	474.5	0.90

472	1991	San Cristobal Amoltepec	fava	vitamin b6	474.5	0.63
473	2007	San Cristobal Amoltepec	fava	vitamin b6	474.5	0.47
474	2018	San Cristobal Amoltepec	fava	vitamin b6	474.5	0.62
475	1991	Santa Catarina Tayata	sheep	vitamin b6	474.5	5.27
476	2007	Santa Catarina Tayata	sheep	vitamin b6	474.5	8.64
477	2018	Santa Catarina Tayata	sheep	vitamin b6	474.5	11.90
478	1991	San Cristobal Amoltepec	sheep	vitamin b6	474.5	1.77
479	2007	San Cristobal Amoltepec	sheep	vitamin b6	474.5	2.50
480	2018	San Cristobal Amoltepec	sheep	vitamin b6	474.5	10.03
481	1991	Santa Catarina Tayata	blue tortilla	vitamin b2	438	9.33
482	2007	Santa Catarina Tayata	blue tortilla	vitamin b2	438	15.17
483	2018	Santa Catarina Tayata	blue tortilla	vitamin b2	438	21.50
484	1991	San Cristobal Amoltepec	blue tortilla	vitamin b2	438	17.49
485	2007	San Cristobal Amoltepec	blue tortilla	vitamin b2	438	10.71
486	2018	San Cristobal Amoltepec	blue tortilla	vitamin b2	438	14.45
487	1991	Santa Catarina Tayata	white tortilla	vitamin b2	438	50.58
488	2007	Santa Catarina Tayata	white tortilla	vitamin b2	438	82.25
489	2018	Santa Catarina Tayata	white tortilla	vitamin b2	438	116.59
490	1991	San Cristobal Amoltepec	white tortilla	vitamin b2	438	20.21
491	2007	San Cristobal Amoltepec	white tortilla	vitamin b2	438	12.38
492	2018	San Cristobal Amoltepec	white tortilla	vitamin b2	438	16.71
493	1991	Santa Catarina Tayata	yellow tortilla	vitamin b2	438	22.47
494	2007	Santa Catarina Tayata	yellow tortilla	vitamin b2	438	36.53
495	2018	Santa Catarina Tayata	yellow tortilla	vitamin b2	438	51.79
496	1991	San Cristobal Amoltepec	yellow tortilla	vitamin b2	438	21.19
497	2007	San Cristobal Amoltepec	yellow tortilla	vitamin b2	438	12.97
498	2018	San Cristobal Amoltepec	yellow tortilla	vitamin b2	438	17.51
499	1991	Santa Catarina Tayata	bean	vitamin b2	438	82.29
500	2007	Santa Catarina Tayata	bean	vitamin b2	438	88.53
501	2018	Santa Catarina Tayata	bean	vitamin b2	438	62.46
502	1991	San Cristobal Amoltepec	bean	vitamin b2	438	7.82
503	2007	San Cristobal Amoltepec	bean	vitamin b2	438	17.50
504	2018	San Cristobal Amoltepec	bean	vitamin b2	438	19.84
505	1991	Santa Catarina Tayata	squash flesh	vitamin b2	438	4.21
506	2007	Santa Catarina Tayata	squash flesh	vitamin b2	438	5.24
507	2018	Santa Catarina Tayata	squash flesh	vitamin b2	438	3.99
508	1991	San Cristobal Amoltepec	squash flesh	vitamin b2	438	0.69
509	2007	San Cristobal Amoltepec	squash flesh	vitamin b2	438	0.51
510	2018	San Cristobal Amoltepec	squash flesh	vitamin b2	438	0.67
511	1991	Santa Catarina Tayata	squash seeds	vitamin b2	438	0.90
512	2007	Santa Catarina Tayata	squash seeds	vitamin b2	438	1.12
513	2018	Santa Catarina Tayata	squash seeds	vitamin b2	438	0.85
514	1991	San Cristobal Amoltepec	squash seeds	vitamin b2	438	0.15

515	2007	San Cristobal Amoltepec	squash seeds	vitamin b2	438	0.11
516	2018	San Cristobal Amoltepec	squash seeds	vitamin b2	438	0.14
517	1991	Santa Catarina Tayata	fava	vitamin b2	438	0.91
518	2007	Santa Catarina Tayata	fava	vitamin b2	438	1.14
519	2018	Santa Catarina Tayata	fava	vitamin b2	438	0.87
520	1991	San Cristobal Amoltepec	fava	vitamin b2	438	0.61
521	2007	San Cristobal Amoltepec	fava	vitamin b2	438	0.45
522	2018	San Cristobal Amoltepec	fava	vitamin b2	438	0.59
523	1991	Santa Catarina Tayata	sheep	vitamin b2	438	9.67
524	2007	Santa Catarina Tayata	sheep	vitamin b2	438	15.84
525	2018	Santa Catarina Tayata	sheep	vitamin b2	438	21.82
526	1991	San Cristobal Amoltepec	sheep	vitamin b2	438	3.25
527	2007	San Cristobal Amoltepec	sheep	vitamin b2	438	4.58
528	2018	San Cristobal Amoltepec	sheep	vitamin b2	438	18.39
529	1991	Santa Catarina Tayata	blue tortilla	vitamin b3	5475	17.12
530	2007	Santa Catarina Tayata	blue tortilla	vitamin b3	5475	27.83
531	2018	Santa Catarina Tayata	blue tortilla	vitamin b3	5475	39.45
532	1991	San Cristobal Amoltepec	blue tortilla	vitamin b3	5475	32.09
533	2007	San Cristobal Amoltepec	blue tortilla	vitamin b3	5475	19.65
534	2018	San Cristobal Amoltepec	blue tortilla	vitamin b3	5475	26.53
535	1991	Santa Catarina Tayata	white tortilla	vitamin b3	5475	60.70
536	2007	Santa Catarina Tayata	white tortilla	vitamin b3	5475	98.70
537	2018	Santa Catarina Tayata	white tortilla	vitamin b3	5475	139.90
538	1991	San Cristobal Amoltepec	white tortilla	vitamin b3	5475	24.25
539	2007	San Cristobal Amoltepec	white tortilla	vitamin b3	5475	14.85
540	2018	San Cristobal Amoltepec	white tortilla	vitamin b3	5475	20.05
541	1991	Santa Catarina Tayata	yellow tortilla	vitamin b3	5475	26.96
542	2007	Santa Catarina Tayata	yellow tortilla	vitamin b3	5475	43.84
543	2018	Santa Catarina Tayata	yellow tortilla	vitamin b3	5475	62.14
544	1991	San Cristobal Amoltepec	yellow tortilla	vitamin b3	5475	25.42
545	2007	San Cristobal Amoltepec	yellow tortilla	vitamin b3	5475	15.57
546	2018	San Cristobal Amoltepec	yellow tortilla	vitamin b3	5475	21.01
547	1991	Santa Catarina Tayata	bean	vitamin b3	5475	29.28
548	2007	Santa Catarina Tayata	bean	vitamin b3	5475	31.50
549	2018	Santa Catarina Tayata	bean	vitamin b3	5475	22.22
550	1991	San Cristobal Amoltepec	bean	vitamin b3	5475	2.78
551	2007	San Cristobal Amoltepec	bean	vitamin b3	5475	6.23
552	2018	San Cristobal Amoltepec	bean	vitamin b3	5475	7.06
553	1991	Santa Catarina Tayata	squash flesh	vitamin b3	5475	4.21
554	2007	Santa Catarina Tayata	squash flesh	vitamin b3	5475	5.24
555	2018	Santa Catarina Tayata	squash flesh	vitamin b3	5475	3.99
556	1991	San Cristobal Amoltepec	squash flesh	vitamin b3	5475	0.69
557	2007	San Cristobal Amoltepec	squash flesh	vitamin b3	5475	0.51

558	2018	San Cristobal Amoltepec	squash flesh	vitamin b3	5475	0.67
559	1991	Santa Catarina Tayata	squash seeds	vitamin b3	5475	2.13
560	2007	Santa Catarina Tayata	squash seeds	vitamin b3	5475	2.65
561	2018	Santa Catarina Tayata	squash seeds	vitamin b3	5475	2.02
562	1991	San Cristobal Amoltepec	squash seeds	vitamin b3	5475	0.35
563	2007	San Cristobal Amoltepec	squash seeds	vitamin b3	5475	0.26
564	2018	San Cristobal Amoltepec	squash seeds	vitamin b3	5475	0.34
565	1991	Santa Catarina Tayata	fava	vitamin b3	5475	0.63
566	2007	Santa Catarina Tayata	fava	vitamin b3	5475	0.78
567	2018	Santa Catarina Tayata	fava	vitamin b3	5475	0.59
568	1991	San Cristobal Amoltepec	fava	vitamin b3	5475	0.42
569	2007	San Cristobal Amoltepec	fava	vitamin b3	5475	0.31
570	2018	San Cristobal Amoltepec	fava	vitamin b3	5475	0.41
571	1991	Santa Catarina Tayata	sheep	vitamin b3	5475	21.45
572	2007	Santa Catarina Tayata	sheep	vitamin b3	5475	35.14
573	2018	Santa Catarina Tayata	sheep	vitamin b3	5475	48.40
574	1991	San Cristobal Amoltepec	sheep	vitamin b3	5475	7.20
575	2007	San Cristobal Amoltepec	sheep	vitamin b3	5475	10.15
576	2018	San Cristobal Amoltepec	sheep	vitamin b3	5475	40.78
577	1991	Santa Catarina Tayata	blue tortilla	vitamin b9	146000	0.00
578	2007	Santa Catarina Tayata	blue tortilla	vitamin b9	146000	0.00
579	2018	Santa Catarina Tayata	blue tortilla	vitamin b9	146000	0.00
580	1991	San Cristobal Amoltepec	blue tortilla	vitamin b9	146000	0.00
581	2007	San Cristobal Amoltepec	blue tortilla	vitamin b9	146000	0.00
582	2018	San Cristobal Amoltepec	blue tortilla	vitamin b9	146000	0.00
583	1991	Santa Catarina Tayata	white tortilla	vitamin b9	146000	0.00
584	2007	Santa Catarina Tayata	white tortilla	vitamin b9	146000	0.00
585	2018	Santa Catarina Tayata	white tortilla	vitamin b9	146000	0.00
586	1991	San Cristobal Amoltepec	white tortilla	vitamin b9	146000	0.00
587	2007	San Cristobal Amoltepec	white tortilla	vitamin b9	146000	0.00
588	2018	San Cristobal Amoltepec	white tortilla	vitamin b9	146000	0.00
589	1991	Santa Catarina Tayata	yellow tortilla	vitamin b9	146000	0.00
590	2007	Santa Catarina Tayata	yellow tortilla	vitamin b9	146000	0.00
591	2018	Santa Catarina Tayata	yellow tortilla	vitamin b9	146000	0.00
592	1991	San Cristobal Amoltepec	yellow tortilla	vitamin b9	146000	0.00
593	2007	San Cristobal Amoltepec	yellow tortilla	vitamin b9	146000	0.00
594	2018	San Cristobal Amoltepec	yellow tortilla	vitamin b9	146000	0.00
595	1991	Santa Catarina Tayata	bean	vitamin b9	146000	243.20
596	2007	Santa Catarina Tayata	bean	vitamin b9	146000	261.65
597	2018	Santa Catarina Tayata	bean	vitamin b9	146000	184.58
598	1991	San Cristobal Amoltepec	bean	vitamin b9	146000	23.11
599	2007	San Cristobal Amoltepec	bean	vitamin b9	146000	51.72
600	2018	San Cristobal Amoltepec	bean	vitamin b9	146000	58.63

601	1991	Santa Catarina Tayata	squash flesh	vitamin b9	146000	5.05
602	2007	Santa Catarina Tayata	squash flesh	vitamin b9	146000	6.29
603	2018	Santa Catarina Tayata	squash flesh	vitamin b9	146000	4.79
604	1991	San Cristobal Amoltepec	squash flesh	vitamin b9	146000	0.82
605	2007	San Cristobal Amoltepec	squash flesh	vitamin b9	146000	0.62
606	2018	San Cristobal Amoltepec	squash flesh	vitamin b9	146000	0.81
607	1991	Santa Catarina Tayata	squash seeds	vitamin b9	146000	0.00
608	2007	Santa Catarina Tayata	squash seeds	vitamin b9	146000	0.00
609	2018	Santa Catarina Tayata	squash seeds	vitamin b9	146000	0.00
610	1991	San Cristobal Amoltepec	squash seeds	vitamin b9	146000	0.00
611	2007	San Cristobal Amoltepec	squash seeds	vitamin b9	146000	0.00
612	2018	San Cristobal Amoltepec	squash seeds	vitamin b9	146000	0.00
613	1991	Santa Catarina Tayata	fava	vitamin b9	146000	0.00
614	2007	Santa Catarina Tayata	fava	vitamin b9	146000	0.00
615	2018	Santa Catarina Tayata	fava	vitamin b9	146000	0.00
616	1991	San Cristobal Amoltepec	fava	vitamin b9	146000	0.00
617	2007	San Cristobal Amoltepec	fava	vitamin b9	146000	0.00
618	2018	San Cristobal Amoltepec	fava	vitamin b9	146000	0.00
619	1991	Santa Catarina Tayata	sheep	vitamin b9	146000	0.00
620	2007	Santa Catarina Tayata	sheep	vitamin b9	146000	0.00
621	2018	Santa Catarina Tayata	sheep	vitamin b9	146000	0.00
622	1991	San Cristobal Amoltepec	sheep	vitamin b9	146000	0.00
623	2007	San Cristobal Amoltepec	sheep	vitamin b9	146000	0.00
624	2018	San Cristobal Amoltepec	sheep	vitamin b9	146000	0.00
625	1991	Santa Catarina Tayata	blue tortilla	vitamin b12	876	0.00
626	2007	Santa Catarina Tayata	blue tortilla	vitamin b12	876	0.00
627	2018	Santa Catarina Tayata	blue tortilla	vitamin b12	876	0.00
628	1991	San Cristobal Amoltepec	blue tortilla	vitamin b12	876	0.00
629	2007	San Cristobal Amoltepec	blue tortilla	vitamin b12	876	0.00
630	2018	San Cristobal Amoltepec	blue tortilla	vitamin b12	876	0.00
631	1991	Santa Catarina Tayata	white tortilla	vitamin b12	876	0.00
632	2007	Santa Catarina Tayata	white tortilla	vitamin b12	876	0.00
633	2018	Santa Catarina Tayata	white tortilla	vitamin b12	876	0.00
634	1991	San Cristobal Amoltepec	white tortilla	vitamin b12	876	0.00
635	2007	San Cristobal Amoltepec	white tortilla	vitamin b12	876	0.00
636	2018	San Cristobal Amoltepec	white tortilla	vitamin b12	876	0.00
637	1991	Santa Catarina Tayata	yellow tortilla	vitamin b12	876	0.00
638	2007	Santa Catarina Tayata	yellow tortilla	vitamin b12	876	0.00
639	2018	Santa Catarina Tayata	yellow tortilla	vitamin b12	876	0.00
640	1991	San Cristobal Amoltepec	yellow tortilla	vitamin b12	876	0.00
641	2007	San Cristobal Amoltepec	yellow tortilla	vitamin b12	876	0.00
642	2018	San Cristobal Amoltepec	yellow tortilla	vitamin b12	876	0.00
643	1991	Santa Catarina Tayata	bean	vitamin b12	876	0.00

644	2007	Santa Catarina Tayata	bean	vitamin b12	876	0.00
645	2018	Santa Catarina Tayata	bean	vitamin b12	876	0.00
646	1991	San Cristobal Amoltepec	bean	vitamin b12	876	0.00
647	2007	San Cristobal Amoltepec	bean	vitamin b12	876	0.00
648	2018	San Cristobal Amoltepec	bean	vitamin b12	876	0.00
649	1991	Santa Catarina Tayata	squash flesh	vitamin b12	876	0.00
650	2007	Santa Catarina Tayata	squash flesh	vitamin b12	876	0.00
651	2018	Santa Catarina Tayata	squash flesh	vitamin b12	876	0.00
652	1991	San Cristobal Amoltepec	squash flesh	vitamin b12	876	0.00
653	2007	San Cristobal Amoltepec	squash flesh	vitamin b12	876	0.00
654	2018	San Cristobal Amoltepec	squash flesh	vitamin b12	876	0.00
655	1991	Santa Catarina Tayata	squash seeds	vitamin b12	876	0.00
656	2007	Santa Catarina Tayata	squash seeds	vitamin b12	876	0.00
657	2018	Santa Catarina Tayata	squash seeds	vitamin b12	876	0.00
658	1991	San Cristobal Amoltepec	squash seeds	vitamin b12	876	0.00
659	2007	San Cristobal Amoltepec	squash seeds	vitamin b12	876	0.00
660	2018	San Cristobal Amoltepec	squash seeds	vitamin b12	876	0.00
661	1991	Santa Catarina Tayata	fava	vitamin b12	876	0.00
662	2007	Santa Catarina Tayata	fava	vitamin b12	876	0.00
663	2018	Santa Catarina Tayata	fava	vitamin b12	876	0.00
664	1991	San Cristobal Amoltepec	fava	vitamin b12	876	0.00
665	2007	San Cristobal Amoltepec	fava	vitamin b12	876	0.00
666	2018	San Cristobal Amoltepec	fava	vitamin b12	876	0.00
667	1991	Santa Catarina Tayata	sheep	vitamin b12	876	52.52
668	2007	Santa Catarina Tayata	sheep	vitamin b12	876	86.04
669	2018	Santa Catarina Tayata	sheep	vitamin b12	876	118.51
670	1991	San Cristobal Amoltepec	sheep	vitamin b12	876	17.63
671	2007	San Cristobal Amoltepec	sheep	vitamin b12	876	24.85
672	2018	San Cristobal Amoltepec	sheep	vitamin b12	876	99.87
