# Supplementary material for: The importance of the traditional milpa in food security and nutritional self-sufficiency in the highlands of Oaxaca, Mexico
Source: PLoS One. 2021 Feb 19;16(2):e0246281. doi: 10.1371/journal.pone.0246281 (PMC7894926; doi:10.1371/journal.pone.0246281)
Supplement: S4 Table — (PDF) [file pone.0246281.s004.pdf]

	municipality	population size	nutrient	number of persons served
1	Santa Catarina Tayata	649	Ca	1866.326904
2	San Cristóbal Amoltepec	889	Ca	663.3932877
3	Santa Catarina Tayata	649	Dietary energy	2303.306155
4	San Cristóbal Amoltepec	889	Dietary energy	895.096621
5	Santa Catarina Tayata	649	Fe	2652.084018
6	San Cristóbal Amoltepec	889	Fe	1151.116438
7	Santa Catarina Tayata	649	Mg	3809.705117
8	San Cristóbal Amoltepec	889	Mg	1483.634105
9	Santa Catarina Tayata	649	P	4347.763836
10	San Cristóbal Amoltepec	889	P	1942.072016
11	Santa Catarina Tayata	649	Protein	3784.466291
12	San Cristóbal Amoltepec	889	Protein	1708.016116
13	Santa Catarina Tayata	649	Vit. A	81.23318113
14	San Cristóbal Amoltepec	889	Vit. A	124.8876712
15	Santa Catarina Tayata	649	Vit. B2	2984.607306
16	San Cristóbal Amoltepec	889	Vit. B2	1305.742009
17	Santa Catarina Tayata	649	Vit. B3	2534.690995
18	San Cristóbal Amoltepec	889	Vit. B3	1193.620274
19	Santa Catarina Tayata	649	Vit. B6	4165.10432
20	San Cristóbal Amoltepec	889	Vit. B6	1607.428872
21	Santa Catarina Tayata	649	Vit. B9	4181.094384
22	San Cristóbal Amoltepec	889	Vit. B9	1961.241781
23	Santa Catarina Tayata	649	Vit. B12	785.7534247
24	San Cristóbal Amoltepec	889	Vit. B12	1002.65411
25	Santa Catarina Tayata	649	Vit. C	14.35739726
26	San Cristóbal Amoltepec	889	Vit. C	17.24640411
27	Santa Catarina Tayata	649	Zn	1572.753699
28	San Cristóbal Amoltepec	889	Zn	950.1942466
