# Supplementary material for: The importance of the traditional milpa in food security and nutritional self-sufficiency in the highlands of Oaxaca, Mexico
Source: PLoS One. 2021 Feb 19;16(2):e0246281. doi: 10.1371/journal.pone.0246281 (PMC7894926; doi:10.1371/journal.pone.0246281)
Supplement: S5 Table — (PDF) [file pone.0246281.s005.pdf]

	municipality	Cropping system	Nutrient/vitamin	Number of persons servec
1	San Cristobal Amoltepec	milpa	Calories	1.881
2	Santa Catarina Tayata	milpa	Calories	4.244
3	San Cristobal Amoltepec	maize	Calories	1.428
4	Santa Catarina Tayata	maize	Calories	3.528
5	San Cristobal Amoltepec	bean	Calories	0.585
6	Santa Catarina Tayata	bean	Calories	0.723
7	San Cristobal Amoltepec	milpa	Protein	2.433
8	Santa Catarina Tayata	milpa	Protein	5.047
9	San Cristobal Amoltepec	maize	Protein	1.458
10	Santa Catarina Tayata	maize	Protein	3.601
11	San Cristobal Amoltepec	bean	Protein	1.500
12	Santa Catarina Tayata	bean	Protein	1.854
13	San Cristobal Amoltepec	milpa	Calcium	1.380
14	Santa Catarina Tayata	milpa	Calcium	3.119
15	San Cristobal Amoltepec	maize	Calcium	1.056
16	Santa Catarina Tayata	maize	Calcium	2.610
17	San Cristobal Amoltepec	bean	Calcium	0.452
18	Santa Catarina Tayata	bean	Calcium	0.558
19	San Cristobal Amoltepec	milpa	Phosphorus	3.187
20	Santa Catarina Tayata	milpa	Phosphorus	6.432
21	San Cristobal Amoltepec	maize	Phosphorus	1.509
22	Santa Catarina Tayata	maize	Phosphorus	3.728
23	San Cristobal Amoltepec	bean	Phosphorus	1.998
24	Santa Catarina Tayata	bean	Phosphorus	2.469
25	San Cristobal Amoltepec	milpa	Iron	1.783
26	Santa Catarina Tayata	milpa	Iron	3.549
27	San Cristobal Amoltepec	maize	Iron	0.869
28	Santa Catarina Tayata	maize	Iron	2.148
29	San Cristobal Amoltepec	bean	Iron	1.329
30	Santa Catarina Tayata	bean	Iron	1.643
31	San Cristobal Amoltepec	milpa	Magnesium	3.602
32	Santa Catarina Tayata	milpa	Magnesium	8.060
33	San Cristobal Amoltepec	maize	Magnesium	2.365

34	Santa Catarina Tayata	maize	Magnesium	5.843
35	San Cristobal Amoltepec	bean	Magnesium	0.509
36	Santa Catarina Tayata	bean	Magnesium	0.629
37	San Cristobal Amoltepec	milpa	Zinc	1.134
38	Santa Catarina Tayata	milpa	Zinc	2.094
39	San Cristobal Amoltepec	maize	Zinc	0.261
40	Santa Catarina Tayata	maize	Zinc	0.644
41	San Cristobal Amoltepec	bean	Zinc	0.859
42	Santa Catarina Tayata	bean	Zinc	1.062
43	San Cristobal Amoltepec	milpa	Vit..A	0.976
44	Santa Catarina Tayata	milpa	Vit..A	1.952
45	San Cristobal Amoltepec	maize	Vit..A	0.000
46	Santa Catarina Tayata	maize	Vit..A	0.000
47	San Cristobal Amoltepec	bean	Vit..A	0.000
48	Santa Catarina Tayata	bean	Vit..A	0.000
49	San Cristobal Amoltepec	milpa	Vit..C	1.047
50	Santa Catarina Tayata	milpa	Vit..C	2.081
51	San Cristobal Amoltepec	maize	Vit..C	0.000
52	Santa Catarina Tayata	maize	Vit..C	0.000
53	San Cristobal Amoltepec	bean	Vit..C	0.037
54	Santa Catarina Tayata	bean	Vit..C	0.046
55	San Cristobal Amoltepec	milpa	Vit..B6	2.880
56	Santa Catarina Tayata	milpa	Vit..B6	6.292
57	San Cristobal Amoltepec	maize	Vit..B6	2.006
58	Santa Catarina Tayata	maize	Vit..B6	4.957
59	San Cristobal Amoltepec	bean	Vit..B6	1.374
60	Santa Catarina Tayata	bean	Vit..B6	1.698
61	San Cristobal Amoltepec	milpa	Vit..B2	1.877
62	Santa Catarina Tayata	milpa	Vit..B2	3.869
63	San Cristobal Amoltepec	maize	Vit..B2	1.087
64	Santa Catarina Tayata	maize	Vit..B2	2.685
65	San Cristobal Amoltepec	bean	Vit..B2	1.320
66	Santa Catarina Tayata	bean	Vit..B2	1.631
67	San Cristobal Amoltepec	milpa	Vit..B3	1.842

**68**	Santa Catarina Tayata	milpa	Vit..B3	4.132
**69**	San Cristobal Amoltepec	maize	Vit..B3	1.304
**70**	Santa Catarina Tayata	maize	Vit..B3	3.222
**71**	San Cristobal Amoltepec	bean	Vit..B3	0.470
**72**	Santa Catarina Tayata	bean	Vit..B3	0.580
**73**	San Cristobal Amoltepec	milpa	Vit..B9	1.514
**74**	Santa Catarina Tayata	milpa	Vit..B9	2.077
**75**	San Cristobal Amoltepec	maize	Vit..B9	0.000
**76**	Santa Catarina Tayata	maize	Vit..B9	0.000
**77**	San Cristobal Amoltepec	bean	Vit..B9	3.901
**78**	Santa Catarina Tayata	bean	Vit..B9	4.820
