# Supplementary material for: The importance of the traditional milpa in food security and nutritional self-sufficiency in the highlands of Oaxaca, Mexico
Source: PLoS One. 2021 Feb 19;16(2):e0246281. doi: 10.1371/journal.pone.0246281 (PMC7894926; doi:10.1371/journal.pone.0246281)
Supplement: S6 Table — (PDF) [file pone.0246281.s006.pdf]

| Municipality | Household type | Fimily size | Migration of family members |
|---|---|---|---|
| Santa Catarina Tayata | Livestock | 3.8 | 0.8 |
| Santa Catarina Tayata | Crop production | 4.1 | 0.5 |
| Santa Catarina Tayata | Migration | 2.1 | 4.1 |
| Santa Catarina Tayata | Off-farm | 5.9 | 0.4 |
| San Cristóbal Amoltepec | Off-farm | 4.0 | 0.3 |
| San Cristóbal Amoltepec | Diversified | 2.9 | 0.8 |
| San Cristóbal Amoltepec | Government support-dependent | 4.0 | 0.2 |

| Months of self-sufficiency | Contribution of off-farm activities to total income (%) | Contribution of agricultural activities to total income (%) | Contribution of livestock activities to total income (%) |
|---|---|---|---|
| 9 | 14.63 | 2.68 | 33.97 |
| 11 | 51.47 | 19.86 | 7.30 |
| 12 | 42.41 | 2.67 | 15.79 |
| 9 | 94.01 | 0.00 | 0.00 |
| 11 | 84.68 | 0.00 | 0.97 |
| 10 | 64.92 | 1.47 | 1.01 |
| 10 | 26.80 | 2.75 | 1.95 |

| Contribution of government support to total income (%) | Contribution of remittances to total income (%) | Total income in Mexican pesos | Squash surface (ha) | Common bean surface (ha) |
|---|---|---|---|---|
| 40.68 | 8.05 | 17690 | 0.00 | 0.65 |
| 19.71 | 1.65 | 96315 | 0.46 | 1.88 |
| 19.45 | 19.68 | 59138 | 0.59 | 1.17 |
| 5.99 | 0.00 | 54871 | 0.07 | 0.40 |
| 9.68 | 4.67 | 22450 | 0.00 | 0.13 |
| 31.81 | 0.79 | 17731 | 0.00 | 1.18 |
| 66.78 | 1.72 | 30994 | 0.17 | 0.75 |

| Fava bean surface (ha) | Maize surface (ha) | Squash yield (t/ha) | Bean yield (t/ ha) | Fava bean yield (t/ha) | Maize yield (t/ha) | TLU |
|---|---|---|---|---|---|---|
| 0.05 | 1.69 | 0.013 | 0.442 | 0.002 | 1.509 | 4.82 |
| 0.18 | 2.42 | 0.041 | 0.678 | 0.015 | 1.741 | 1.99 |
| 0.22 | 1.96 | 0.021 | 0.323 | 0.006 | 1.233 | 2.24 |
| 0.07 | 1.06 | 0.014 | 0.338 | 0.000 | 1.786 | 0.98 |
| 0.00 | 0.54 | 0.000 | 0.068 | 0.000 | 0.381 | 0.38 |
| 0.18 | 1.64 | 0.000 | 0.145 | 0.053 | 0.661 | 1.28 |
| 0.19 | 0.79 | 0.000 | 0.135 | 0.002 | 0.485 | 0.42 |
